# Morphological and molecular affinities of two East Asian species of *Stenhelia* (Crustacea, Copepoda, Harpacticoida)

**DOI:** 10.3897/zookeys.411.7346

**Published:** 2014-05-28

**Authors:** Tomislav Karanovic, Kichoon Kim, Wonchoel Lee

**Affiliations:** 1 Hanyang University, Department of Life Sciences, Seoul 133-791, Korea; 2 University of Tasmania, Institute for Marine and Antarctic Studies, Hobart, Tasmania 7001, Australia

**Keywords:** Miraciidae, Stenheliinae, marine, systematics, phylogeny, DNA barcoding

## Abstract

Definition of monophyletic supraspecific units in the harpacticoid subfamily Stenheliinae Brady, 1880 has been considered problematic and hindered by the lack of molecular or morphology based phylogenies, as well as by incomplete original descriptions of many species. Presence of a modified seta on the fifth leg endopod has been suggested recently as a synapomorphy of eight species comprising the redefined genus *Stenhelia* Boeck, 1865, although its presence was not known in *S. pubescens* Chislenko, 1978. We redescribe this species in detail here, based on our freshly collected topotypes from the Russian Far East. The other species redescribed in this paper was collected from the southern coast of South Korea and identified as the Chinese *S. taiae* Mu & Huys, 2002, which represents its second record ever and the first one in Korea. A fragment of the mtCOI gene was successfully PCR-amplified from two specimens of each species, which represents the first molecular data for this genus, and from additional 19 specimens belonging to six different species of other stenheliins from Korea and Russia. Reconstructed phylogenies confirm previously postulated monophyly of *Stenhelia* and polyphyly of the closely related genus *Delavalia* Brady, 1869. Average pairwise maximum likelihood distances between *S. pubescens* and *S. taiae* are only slightly above 10%, suggesting a very close relationship despite numerous newly discovered micro-morphological differences and despite macro-morphological similarities being probable plesiomorphies.

## Introduction

The subfamily Stenheliinae Brady, 1880 is currently recognised as one of three well-defined suprageneric groups within the second largest harpacticoid family Miraciidae Dana, 1846, beside the nominotypical subfamily and Diosaccinae Sars, 1906 (see Willen 2000; Boxshall and Halsey 2004; Wells 2007; Huys and Mu 2008). Stenheliins are common inhabitants of the marine benthos, and can be found from the deep sea (Willen 2003) to shallow brackish waters (Dussart and Defaye 2001). Although there is some disagreement about the exact number of morphological synapomorphies defining this subfamily (Willen 2000, 2002; Huys and Mu 2008), these six are undisputed for adults: laterally displaced genital apertures in females; triangular and usually bifid rostrum, with dorsal pair of sensilla inserted in deep anterior recesses; elongated basis and endopod of mandibula (often also with one extremely long and strong seta); maxilliped with only three syncoxal setae, closely positioned to one another, and setation of the ancestral second endopodal segment lost; female fifth leg with laterally directed exopod; and some form of sexual dimorphism in the second leg (although probably secondarily lost in several species). Some additional synapomorphies are postulated for their naupliar morphology (Dahms and Bresciani 1993, Dahms et al. 2005) but they need to be verified in a broader taxon sampling (Huys and Mu 2008). Ninety-three valid stenheliin species (Wells 2007; Walter and Boxshall 2014; Karanovic and Kim 2014) are currently classified into 12 genera: *Anisostenhelia* Mu & Huys, 2002 (monospecific); *Beatricella* T. Scott, 1905 (monospecific); *Cladorostrata* Tai & Song, 1979 (two species); *Delavalia* Brady, 1869 (53 species and subspecies); *Itostenhelia* Karanovic & Kim, 2014 (two species); *Melima* Por, 1964 (six species); *Muohuysia* Ozdikmen, 2009 (monospecific); *Onychostenhelia* Itô, 1979 (two species); *Pseudostenhelia* Wells, 1967 (four species); *Stenhelia* Boeck, 1865 (eight species); *Wellstenhelia* Karanovic & Kim 2014 (eight species), and *Willenstenhelia* Karanovic & Kim, 2014 (five species).

The most speciose and morphologically most diverse genus *Delavalia* is also taxonomically most problematic, and expectedly postulated to be either paraphyletic (Willen 2002) or polyphyletic (Mu and Huys 2002). Several groups of species were recognized in this genus by Willen (2003) and Huys and Mu (2008), mostly based on intuitive methods and without phylogenetic or nomenclatural consideration. Karanovic and Kim (2014) demonstrated the polyphyletic nature of *Delavalia* using molecular phylogenies and erected three new genera for nine new species and six previously described members of *Delavalia*, each supported by molecular data and a number of morphological synapomorphies. The latter authors used two *Stenhelia* species as outgroups in their molecular analyses, which are the subject of this paper.

The genus *Stenhelia* was redefined recently by Mu and Huys (2002) and restricted to a core goup of species formerly allocated to the subgenus *Stenhelia (Stenhelia)*. In addition to the type species, *Stenhelia gibba* Boeck, 1865, the genus currently contains the following seven species: *Stenhelia curviseta* Lang, 1936; *Stenhelia divergens* Nicholls, 1939; *Stenhelia peniculata* Lang, 1965; *Stenhelia proxima* Sars, 1906; *Stenhelia pubescens* Chislenko, 1978; *Stenhelia sheni* Mu & Huys, 2002; and *Stenhelia taiae* Mu & Huys, 2002. Mu and Huys (2002) suggested the presence of a modified seta on the fifth leg endopod as a generic synapomorphy, although its presence was not known in *Stenhelia pubescens*. This prompted us to redescribe this species in detail here, based on our freshly collected topotypes from the Russian Far East (Posyet Bay near Vladivostok). Another species of *Stenhelia* was collected from the southern coast of South Korea and identified as *Stenhelia taiae*, originally described from China, which represents its second record ever and the first one in Korea. Beside detailed redescriptions of these two species, we also provide their mtCOI sequences, which represent the first molecular data for this genus. One of the aims was to test the generic monophyly reconstructing molecular phylogenies in a larger group of stenheliin copepods. We also aimed to test if the two *Stenhelia* species are closely related, as suggested by Mu and Huys (2002) based on the armature of the third leg endopod and the shape of the first leg endopod, because these seem to be in a plesiomorphic state in the two species when compared with other congeners.

Employing molecular techniques in addition to traditional morphological ones was one of the priorities of this study to aid in species delineation and reconstruction of their phylogenetic relationships. Recently, DNA-based species identification methods, referred to as “DNA barcoding”, have been widely employed to estimate levels of species diversity, with the 5’end of the mitochondrial cytochrome C oxidase subunit 1 gene (mtCOI) proposed as the “barcode” for all animal species (Hebert et al. 2003). The advantage of the mtCOI gene is that it often shows low levels of genetic variation within species, but high levels of divergence between species; for the most common divergence values in a variety of crustacean taxa see Lefébure et al. (2006). In recent years several studies on copepods showed that combining molecular and morphological methods can help answer questions related to cryptic speciation (Bláha et al. 2010; Sakaguchi and Ueda 2010; Karanovic and Krajicek 2012a, Hamrova et al. 2012), invasions of new habitats and colonisation pathways (Lee et al. 2003, 2007; Winkler et al. 2008; Karanovic and Cooper 2011a, 2012), anthropogenic translocation (Karanovic and Krajicek 2012a), short range endemism and allopatry (Karanovic and Cooper 2011a), and definition of supraspecific taxa in conservative genera or families (Huys et al. 2006, 2007, 2009, 2012; Wyngaard et al. 2010; Karanovic and Cooper 2011b, Karanovic and Krajicek 2012b, Karanovic and Kim 2014). However, some studies showed that currently prevailing morphological methods of identifying copepod species are inadequate, and suggested the use of alternative microstructures, such as pores and sensilla pattern on somites (Alekseev et al. 2006; Karanovic and Krajicek 2012a; Karanovic and Cho 2012; Karanovic and Kim 2014; Karanovic and Lee 2012; Karanovic et al. 2012, 2013), an approach also tested in this study.

## Material and methods

All Korean samples for this study were taken at seventeen stations in Gwangyang Bay, on the South Coast of South Korea, on four occasions: 18 February 2012, 30 July 2012, 14 October 2012, and 18 November 2012 (see Karanovic and Kim 2014). Depth ranged from four to 11 metres and environmental conditions changed greatly with seasons; those measured on 18 January 2006 are presented in Table 1. We found no correlation between environmental data and distribution of stenheliins. A handheld multiparameter water quality meter YSI556 (YSI Environmental, Yellow Springs, USA) was used for all measurements, except for chlorophyl a, which was measured by manual filtering with different size filters, and temperature, which was measured with a mercury fill glass thermometer. Coordinates were taken with a Garmin GPS, model Oregon 300. Granular analysis of the sediment was conducted manually, following the methods and classification of Folk (1974). Sediment samples were primarily collected with a van Veen grab sampler (surface area: 0.1 m^2^) from the Hansan research vessel. Subsamples were then collected by acrylic corers (surface area: 10 cm^2^) for quantitative analysis, and surface sediments were collected by a small shovel for qualitative analysis. Each sediment sample was fixed in 99.9% ethanol. Animals in the sediments were extracted by Ludox method (Burgess 2001) and preserved in 99.9% ethanol for morphological or molecular studies. Specimens from Posyet Bay (Minonosok inlet) in Russia were collected with hand-nets (100 μm mesh size) using Scuba-diving from a sandy bottom and between four and seven metres of depth, and also fixed in 99.9% ethanol. Locality data and number of specimens are given in the Material examined section for each species below. All material is deposited at the National Institute of Biological Resources (NIBR), Incheon, South Korea.

**Table 1.** Environmental conditions at 17 sampling stations in Gwangyang Bay, rescorded on 18 January 2006. Water temperature was measured on the surface. Granular analysis was conducted manually according to the protocol described by Folk (1974). Abbreviations: WT, water temperature; ST, sediment temperature; Sal., salinity; DO, dissolved oxygen; Cond., conductivity.

Station	Temperature (C)		pH	Sal. (ppt)	DO (mg/L)	Chlorophyl a		Cond. (mS/cm)	Granular analysis			Coordinates
	WT	ST				total	nano		gravel	sand	mud	
St.01	5.9	7.0	8.1	33.3	11.5	4.6	2.2	32.8	0.0%	9.8%	90.2%	34.913194°N, 127.600917°E
St.02	6.3	7.1	8.1	33.3	11.0	4.5	0.6	33.1	0.0%	46.1%	53.9%	34.881861°N, 127.635083°E
St.03	5.1	7.0	7.9	33.4	12.5	5.2	2.9	32.8	1.9%	37.0%	63.0%	34.884417°N, 127.664028°E
St.04	5.1	7.8	8.2	31.8	12.0	3.1	1.5	30.8	0.1%	29.6%	70.4%	34.910722°N, 127.696806°E
St.05	6.0	7.3	7.3	33.4	10.8	8.9	8.9	32.9	0.0%	19.7%	80.3%	34.852500°N, 127.684722°E
St.06	6.3	7.2	8.1	33.3	12.0	4.1	2.0	33.1	0.0%	13.3%	86.7%	34.860861°N, 127.733417°E
St.07	6.4	8.3	8.2	33.4	12.3	6.7	1.4	33.3	0.0%	13.7%	86.3%	34.897056°N, 127.757722°E
St.08	6.8	8.8	8.2	32.2	10.8	3.9	0.3	32.6	0.0%	16.6%	83.4%	34.865417°N, 127.767222°E
St.09	5.9	7.3	7.5	27.1	12.9	-	-	27.2	0.0%	25.4%	74.6%	34.951389°N, 127.734361°E
St.10	5.9	8.1	8.2	29.5	12.8	3.7	0.9	29.4	0.1%	55.1%	44.9%	34.920944°N, 127.785528°E
St.11	7.7	8.1	7.9	33.4	10.1	0.5	0.4	34.4	0.0%	31.0%	69.0%	34.924333°N, 127.852333°E
St.12	5.8	8.3	8.2	30.7	11.5	3.8	0.4	30.4	0.0%	67.0%	33.0%	34.890139°N, 127.795111°E
St.13	6.6	9.2	8.1	33.2	11.5	5.6	1.5	33.3	0.4%	73.3%	26.7%	34.852750°N, 127.791000°E
St.14	6.6	8.1	8.2	33.3	10.9	5.0	3.8	33.3	0.0%	46.6%	53.4%	34.824222°N, 127.787750°E
St.15	6.9	7.7	8.2	33.6	10.8	3.2	1.1	33.9	0.3%	60.5%	39.5%	34.797194°N, 127.786444°E
St.16	6.7	7.5	8.2	33.8	10.9	6.6	3.5	34.0	2.5%	33.7%	66.3%	34.768889°N, 127.783806°E
St.17	6.2	7.7	8.2	33.8	10.5	4.4	1.6	33.5	0.0%	37.0%	63.0%	34.743444°N, 127.778972°E

Specimens were dissected and mounted on microscope slides in Faure’s medium (see Stock and von Vaupel Klein 1996), and dissected appendages were then covered by a coverslip. For the urosome or the entire animal, two human hairs were mounted between the slide and coverslip, so the parts would not be compressed. All line drawings were prepared using a drawing tube attached to a Leica MB2500 phase-interference compound microscope, equipped with N-PLAN (5×, 10×, 20×, 40× and 63× dry) or PL FLUOTAR (100× oil) objectives. Specimens that were not drawn were examined in propylene glycol and, after examination, were again preserved in 99.9% ethanol. Specimens for scanning electron micrography (SEM) were dehydrated in progressive ethanol concentrations, transferred into pure isoamyl-acetate, critical-point dried, mounted on stubs, coated in gold, and observed under a Hitachi S-4700 microscope on the in-lens detector, with an accelerating voltage of 10 kV and working distances between 12.3 and 13.4 mm; micrographs were taken with a digital camera.

Morphological terminology follows Huys and Boxshall (1991), except for the numbering of the setae of the caudal rami and small differences in the spelling of some appendages (antennula, mandibula, maxillula instead of antennule, mandible, maxillule), as an attempt to standardise the terminology for homologous appendages in different crustacean groups. Sensilla and pores on all somites (body segments) were examined in detail, but are not numbered or marked otherwise on the figures. Only the first presented species is described in full, while the subsequent description is shortened by making it comparative.

Specimens for molecular analysis were examined without dissection under a compound microscope (objective 63× dry) in propylene glycol, using a cavity well slide with a central depression. After examination they were returned to 99.9% ethanol. Before amplification whole specimens were transferred into distilled water for two hours for washing (to remove ethanol), and then minced with a small glass stick. DNA was extracted from whole specimens, except in one case when only one antennula was available, using the LaboPass^TM^ extraction kit (COSMO Co. Ltd., Korea) and following the manufacturer’s protocols for fresh tissue, except that samples were incubated in the Proteinase K solution overnight, step five was skipped, and 60 instead of 200 μl of Buffer AE was added in the final step, to increase the density of DNA. Mitochondrial cytochrome oxidase subunit I (mtCOI) gene was amplified through polymerase chain reaction (PCR) using PCR premix (BiONEER Co.) in TaKaRa PCR thermal cycler (Takara Bio Inc., Otsu, Shiga, Japan). The amplification primers used were the ‘universal’ primers LCO1490 and HCO2198 (Folmer et al. 1994). The amplification protocol was: initial denaturation 94 °C for 300 s, 40 cycles of denaturation 94 °C for 30 s, annealing at 42 °C for 120 s, extension at 72 °C for 60 s; final extension at 72 °C for 600 s, and final product was stored at 4 °C. PCR results were checked by electrophoresis of the amplification products on 1% agarose gel with ethidium bromide. PCR products were purified with a LaboPass^TM^ PCR purification kit and sequenced in both directions using a 3730xl DNA analyzer (Macrogen, Korea). For this study, DNA was extracted and the COI fragment successfully PCR amplified from 23 stenheliin specimens (Table 2).

**Table 2.** List of copepod specimens for which mtCOI fragment was successfully amplified.

Code	Species	Country	Station	Date	Bases	GenBank
0330	*Itostenhelia golikovi*	Russia	Posyet Bay	06 May 2012	448	KF524863
0433	*Itostenhelia golikovi*	Russia	Posyet Bay	06 May 2012	515	KF524864
0631	*Itostenhelia golikovi*	Russia	Posyet Bay	06 May 2012	514	KF524865
0734	*Itostenhelia golikovi*	Russia	Posyet Bay	06 May 2012	503	KF524866
0832	*Itostenhelia golikovi*	Russia	Posyet Bay	06 May 2012	493	KF524867
0176	*Itostenhelia polyhymnia*	Korea	10	30 Jul 2012	660	KF524868
0273	*Itostenhelia polyhymnia*	Korea	10	30 Jul 2012	664	KF524869
0271	*Itostenhelia polyhymnia* L-form	Korea	10	30 Jul 2012	278	KF524883
8417	*Schizopera leptafurca*	Australia	YYAC0016A	20 Mar 2010	517	JQ390578
0152	*Stenhelia pubescens*	Russia	Posyet Bay	06 May 2012	659	KF524870
0254	*Stenhelia pubescens*	Russia	Posyet Bay	06 May 2012	647	KF524871
0163	*Stenhelia taiae*	Korea	16	18 Nov 2012	558	KF524884
0167	*Stenhelia taiae*	Korea	16	18 Nov 2012	662	KF524885
0122	*Wellstenhelia calliope*	Korea	5	30 Jul 2012	576	KF524872
0187	*Wellstenhelia clio*	Korea	10	30 Jul 2012	519	KF524873
0113	*Wellstenhelia qingdaoensis*	Korea	15	18 Nov 2012	518	KF524874
0143	*Willenstenhelia thalia*	Korea	10	30 Jul 2012	657	KF524875
0146	*Willenstenhelia thalia*	Korea	10	18 Nov 2012	664	KF524878
0241	*Willenstenhelia thalia*	Korea	10	30 Jul 2012	524	KF524876
0245	*Willenstenhelia thalia*	Korea	10	18 Nov 2012	662	KF524879
0342	*Willenstenhelia thalia*	Korea	10	30 Jul 2012	330	KF524877
0348	*Willenstenhelia thalia*	Korea	10	18 Nov 2012	660	KF524880
0444	*Willenstenhelia thalia*	Korea	10	18 Nov 2012	667	KF524881
0547	*Willenstenhelia thalia*	Korea	10	18 Nov 2012	661	KF524882

Obtained sequences were checked manually and aligned by ClustalW algorithm (Thompson et al. 1994) in MEGA version 5 (Tamura et al. 2011). The alignment was checked again and all sites were unambiguously aligned. The best evolutionary model of nucleotide substitution for our dataset was established by Akaike Information Criterion, performed with jModelTest (Guindon and Gascuel 2003; Posada 2008). For the maximum likelihood (ML) analysis the Hasegawa-Kishino-Yano model (Hasegawa et al. 1985) with gamma distributed rate heterogeneity (HKY + G) was selected. Neighbour joining (NJ) analysis used the Tamura-Nei model (Tamura and Nei 1993) with uniform rates (TN). Maximum parsimony (MP) analysis was conducted using a heuristic search option and default options (TBR branch swapping, ACCTRAN character state optimisation), with the exception of using random stepwise addition repeated 100 times. All phylogenetic and molecular evolutionary analyses were conducted using MEGA version 5 (Tamura et al. 2011). Five hundred bootstrap replicates were performed to obtain a relative measure of node support for the resulting trees. Average pairwise NJ distances for each dataset were also computed in MEGA version 5 using the Tamura-Nei model. All trees were rooted with *Schizopera leptafurca* Karanovic & Cooper, 2012 from Western Australia, its mtCOI sequences also available from GenBank prior to this study [JQ390578.1], which belongs to the subfamily Diosaccinae Sars, 1906 of the family Miraciidae Dana, 1846.

## Systematics

### Subphylum Crustacea Brünich, 1772
Class Maxillopoda Dahl, 1956
Subclass Copepoda H. Milne Edwards, 1840
Order Harpacticoida Dana, 1846
Family Miraciidae Dana, 1846
Subfamily Stenheliinae Brady, 1880
Genus *Stenhelia* Boeck, 1865

#### Stenhelia pubescens

Chislenko, 1978

http://species-id.net/wiki/Stenhelia_pubescens

**Figure 1.**
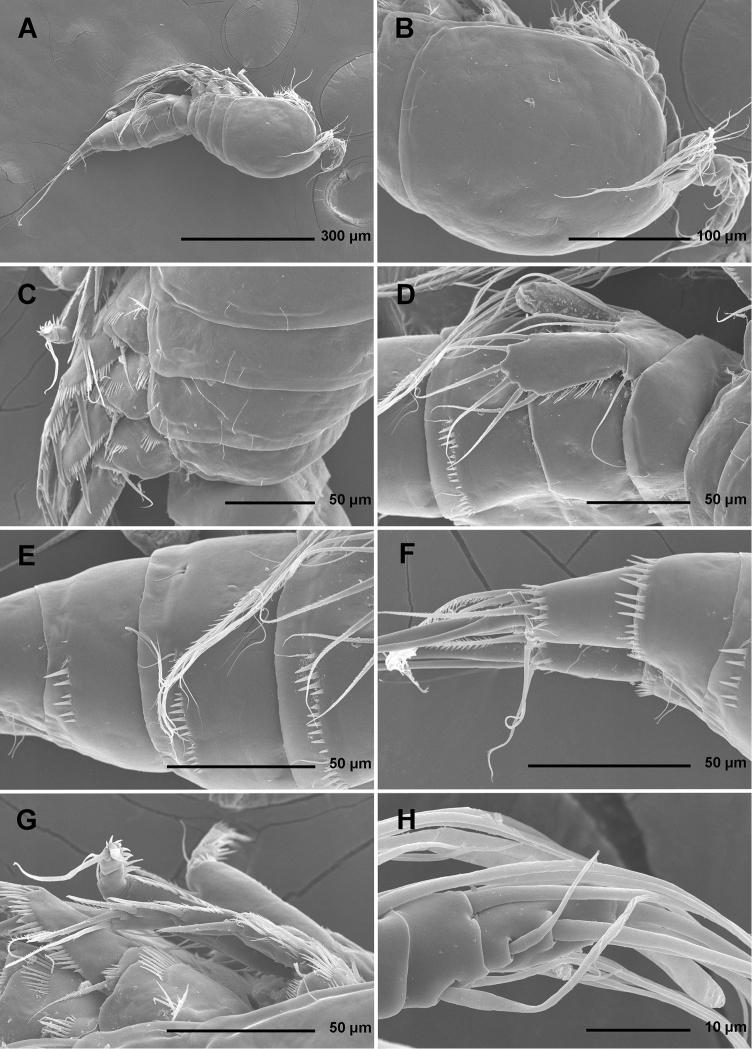
*Stenhelia pubescens* Chislenko, 1978, scanning electron micrographs, female 1: **A** habitus, lateral **B** cephalothorax, lateral **C** free thoracic somites, lateral **D** fifth pedigerous somite and genital double-somite, lateral, with one spermatophore attached on ventral side **E** fourth and fifth urosomites, lateral **F** anal somite and caudal rami, lateral **G** first legs and proximal part of second and third legs, lateral **H** distal part of right antennula, dorsal.

Figs 1 [Fig F2] [Fig F3] [Fig F4] [Fig F5] [Fig F6] –7

##### Synonymy.

*Stenhelia (Stenhelia) pubescens* Chislenko, sp. n. – Chislenko 1978: p. 173, Figs 9–11.

##### Type locality.

Russia, Primorsky Krai, Sea of Japan, Posyet Bay, Minonosok inlet, benthic sands at 3-4 m depth, 42.609258°N, 130.861661°E.

##### Specimens examined.

Two females (one ovigerous) together on one SEM stub (collection number NIBRIV0000232715), one female dissected on one slide (collection number NIBRIV0000232716), one female in ethanol (collection number NIBRIV0000232717), and two ovigerous females destroyed for DNA sequences (GenBank accession nos. KF524870 & KF524871); all from type locality, 6 May 2012, leg. Y. Trebukhova.

##### Redescription of female.

**Figure 2. F2:**
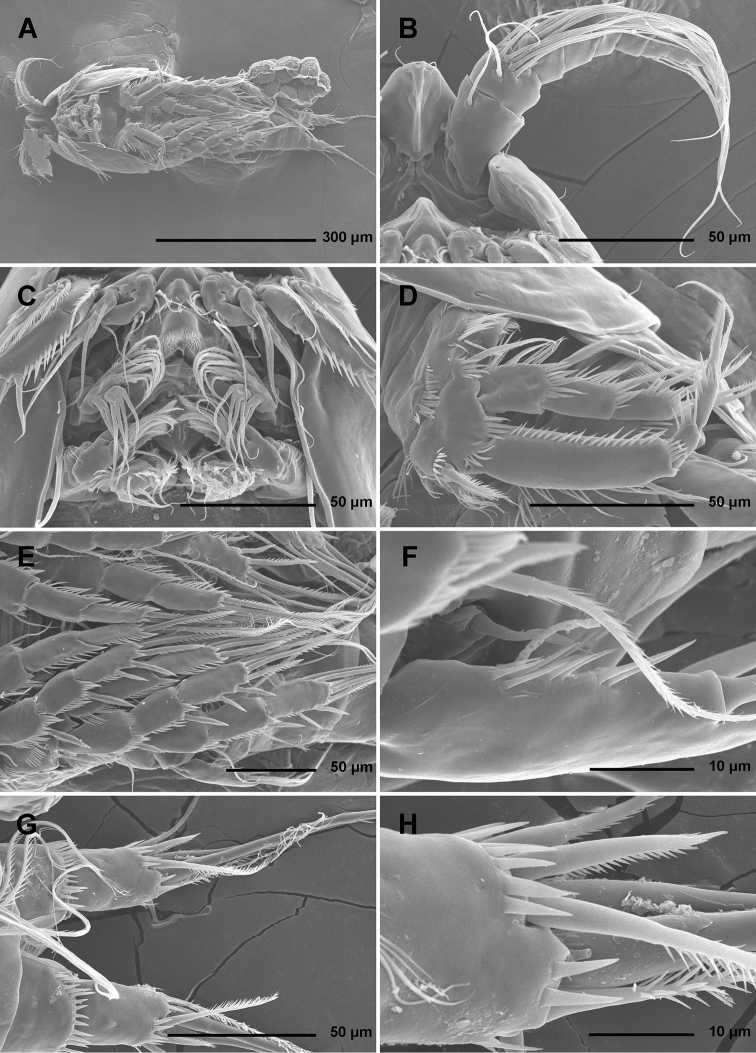
*Stenhelia pubescens* Chislenko, 1978, scanning electron micrographs, ovigerous female 2: **A** habitus, ventral **B** rostrum and left antennula, ventral **C** mouth appendages, ventral **D** first leg, anterior **E** second, third, and fourth legs, anterior **F** exopod of fifth leg and sixth leg, ventral **G** anal somite and caudal rami, ventral **H** posterior part of left caudal ramus, ventral.

Total body length, measured from tip of rostrum to distal margin of caudal rami, from 558 to 583 μm (n = 6). Colour of preserved specimens yellowish; live specimens not observed. Nauplius eye not visible. Several filamentous bacterial colonies in various places, some resembling sensilla (see Fig. 1C). Prosome comprising cephalothorax with completely fused first pedigerous somite, and three free pedigerous somites; urosome comprising first urosomite (= fifth pedigerous somite), genital double-somite (fused genital and third urosomites) and three free urosomites (last one being anal somite). Short sclerotized joint between prosome and urosome only discernible on ventral side. Habitus (Figs 1A, 2A) robust, spindle shaped in dorsal view, widest at posterior end of cephalothorax and tapering posteriorly, boundary between prosome and urosome conspicuous; prosome/urosome length ratio about 1.2, but prosome much wider and more voluminous. Body length/width ratio about 2.9; cephalothorax 1.65 times as wide as genital double-somite. Free pedigerous somites without lateral or dorsal expansions, pleurons only partly covering coxae of legs in lateral view (Fig. 1C). Integument of all somites relatively weakly sclerotized, generally very smooth, without cuticular windows or pits. Hyaline fringe of all somites broad and smooth, except for fourth pedigerous somite with narrow fringe dorsally, and for anal somite without hyaline fringe. Surface ornamentation of somites and caudal rami consisting of three unpaired dorsal pores, 61 paired pores and sensilla, and posterior row of spinules on last four urosomites only.

**Figure 3. F3:**
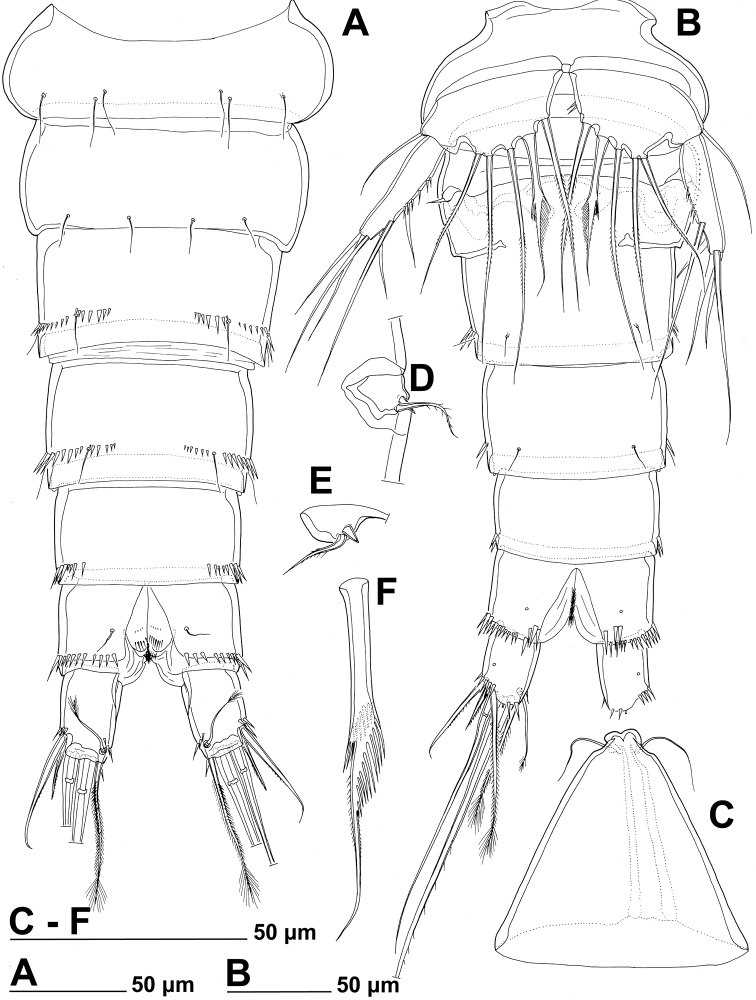
*Stenhelia pubescens* Chislenko, 1978, line drawings, female 3: **A** urosome, dorsal **B** urosome, ventral (armature on left caudal ramus omitted) **C** rostrum, dissected and compressed, dorsal **D** sixth leg, dorso-lateral **E** sixth leg, ventro-lateral **F** fifth leg second endopodal seta from inner side, anterior.

Rostrum (Figs 1B, 2B, 3C) large, trapezoidal, clearly demarcated at base, reaching midlength of second antennular segment, with bilobate tip, about as long as wide, with smooth dorsal surface and central keel on ventral surface, with two large lateral sensilla near tip inserted into deep recesses.

Cephalothorax (Figs 1B, 2A, B, D) tapering anteriorly in dorsal view, about as long as wide; comprising 35% of total body length. Surface of cephalothoracic shield with three pairs of small pores near antero-ventral corner between antennula and antenna (Fig. 2B), one dorsal unpaired pore in anterior half, and 25 pairs of long sensilla (Fig. 1B); of those only eight pairs of sensilla belonging to first pedigerous somite incorporated into cephalothorax (Figs 1B, 2D)

Pleuron of second pedigerous somite (first free) (Fig. 1C) with nearly rectangular lateral section, without pores but with seven pairs of large sensilla, two of them near lateral margin; serial homologies with sensilla on posterior part of cephalothorax (belonging to first pedigerous somite) difficult to define, except perhaps for anterior lateral sensilla and two other posterior pairs.

Pleuron of third pedigerous somite (Fig. 1C) somewhat shorter than that of second pedigerous somite and with slightly more rounded lateral section, but also with no pores and with seven pairs of large sensilla; recognising sensilla serially homologous to those on pleuron of second pedigerous somite easy for all seven pairs.

Pleuron of fourth pedigerous somite (Fig. 1C) much shorter and with more rounded lateral section than those of previous two somites, especially narrow in dorsal view, with only five pairs of large sensilla; serial homology of sensilla to those on two previous somites relatively difficult to establish, but probably two dorsal pairs homologous to two dorsalmost pairs on pleuron of third pedigerous somite and two lateral pairs homologous to those near lateral margin on two previous somites.

First urosomite (Figs 1D, 3A, B) about as long and as wide as fourth pedigerous somite but with wider hyaline fringe, with only three dorso-lateral pairs of long sensilla and no pores or spinules.

Genital double-somite (Figs 1D, 3A, B) about 1.2 times as wide as long (ventral view); completely fused ventrally but with deep suture indicating original segmentation between genital and third urosomites dorso-laterally, thus dividing double-somite into equally long halves; anterior half of genital double-somite 1.2 times as wide as posterior, inflated laterally; anterior part with one unpaired dorsal pore and two pairs of long dorsal sensilla; serially homologous sensilla of anterior part of double-somite and those of first urosomite not easy to establish; posterior part with three pairs of posterior sensilla (one dorsal, one lateral, and one ventral) and long row of posterior dorso-lateral spinules of various length; establishing serially homologous sensilla of posterior and anterior part of double-somite not easy; hyaline fringe wider than in first urosomite. Female genital complex (Fig. 3B) weakly sclerotized and hardly distinguishable from internal sutures and soft tissue, copulatory pores not exposed on surface but their position could be deduced from attached spermatophores (Fig. 1D); paired genital apertures situated ventro-laterally, close to anterior margin and covered by reduced sixth legs.

Third urosomite (Figs 1E, 3A, B) slightly narrower than posterior half of gential double-somite, but about as long and ornamented very similarly with three pairs of posterior sensilla and posterior row of spinules of various size, interrupted dorsally and ventrally; all sensilla with homologous pairs on posterior half of genital double-somite; hyaline fringe as wide as in genital double-somite.

Fourth urosomite (preanal) (Figs 1E, 3A, B) without sensilla or pores, only ornamentation posterior row of spinules with wider dorsal and ventral interruption than in previous two somites; hyaline fringe slightly narrower than in third urosomite.

Fifth urosomite (anal) (Figs 1F, 2G, 3A, B) clefted medially in posterior half, without anal operculum, with one pair of large dorsal sensilla, one pair of ventral pores, and posterior row of spinules at base of each caudal ramus; anal sinus with several diagonal rows of hair-like spinules on both sides of median cleft, widely open, with weakly sclerotised walls, and without chitinous projections.

Caudal rami (Figs 1F, 2G, H, 3A, B) short and slender, cylindrical, about as long as anal somite, 1.5 times as long as wide (dorsal view), slightly divergent, with space between them about one ramus width; armature consisting of seven setae (three lateral, one dorsal and three apical), all in posterior sixth of ramus length; ornamentation consisting of one ventral pore at midlenght, one posterior ventral tubular pore, several spinules at base of each lateral seta and at base of dorsal seta, and two large posterior ventral spinules at base of innermost apical seta. Dorsal seta slender, plumose at distal tip, inserted close to inner margin, about 1.2 times as long as caudal ramus, triarticulate at base (i.e. inserted on two pseudojoints). Lateral setae all bipinnate and uniarticulate; ventralmost one longest and most slender, with distal tuft of longer pinnules, inserted very close to distal margin, about 1.3 times as long as caudal ramus; dorsalmost one strongest, without distal tuft of long pinnules, about 0.8 times as long as ventralmost one, inserted slightly more anteriorly than ventralmost one, at about same level as dorsal seta; central one half as long as dorsalmost one, also strong, inserted at about same level, also without distal tuft of long pinnules. Inner apical seta only slightly shorter than ventralmost lateral seta but very similar in thickness and ornamentation, i.e. also with distal tuft of long pinnules. Principal apical setae not fused basally, both with breaking planes; middle apical seta much stronger and longer, about 2.2 times as long as outer apical one, bipinnate; outer apical seta smooth, about 3.8 times as long as caudal ramus.

Antennula (Figs 1H, 2B, 5A) eight-segmented, joined to cephalotholax with small triangular cuticular plate, about half as long as cephalothorax, with single short anterior row of spinules on first segment. Fourth segment sometimes with suture along caudal margin. Distal caudal corner of first segment not produced. Long aesthetasc on fourth segment slender, fused basally with adjacent large seta, and reaching beyond tip of appendage; slender short apical aesthetasc on eighth segment fused basally with two apical setae, forming apical acrothek. Setal formula: 1.11.9.6+ae.3.4.4.6+ae. All setae smooth, dorsalmost setae on second segment with breaking plane, two caudal setae on seventh segment and four caudal setae on eight segment biarticulate. Length ratio of antennular segments, measured along caudal margin, 1 : 0.4 : 0.3 : 0.4 : 0.3 : 0.4 : 0.4 : 0.5.

Antenna (Figs 2C, 5B, C) relatively short, composed of coxa, allobasis, one-segmented endopod and three-segmented exopod. Coxa short, with arched row of long posterior spinules. Allobasis with smaller or bigger suture marking ancestral division between basis and first endopodal segment, most robust segment of antenna, more than four times as long as coxa and about as long as endopod, widest at base and about 2.5 times as long as wide, with single unipinnate inner seta at about midlength and several longer and smaller spinules in proimal half. Endopod about as wide as distal part of allobasis, almost cylindrical, about 3.6 times as long as wide, with two surface frills subdistally, row of large spinules all along anterior margin, two lateral spines flanking two thin setae, apical armature consisting of seven pinnae setae (four strong, long, and geniculate, innermost one strong but short, and two short and slender); two caudalmost setae fused basally. Exopod long and slender, almost cylindrical, about as long as allobasis but only half as wide; armature formula 1.1.4 and length ratio of segments 1 : 0.3 : 1.1; proximal segment with transverse distal row of small anterior spinules, bearing a unipinnate seta close to distomedial corner; second segment unornamented, with a unipinnate setae at distomedial corner; distal segment with two parallel longitudinal anterior rows of small spinules joining at distal margin, with one bipinnae inner seta, at about first third of its length, and three apical slender (two smooth and one bipinnate).

Labrum (Fig. 2C) large and complex tri-dimensional structure, trapezoidal in anterior view, rigidly sclerotized, with relatively wide convex cutting edge, subapically and apically with several rows of short slender spinules, with one additional transverse row of small anterior spinules and another patch of small posterior spinules.

Paragnaths (Fig. 2C) also forming complex tri-dimensional structure, trilobate, with two ellipsoid anterior lobes and one central, much shorter posterior lobe, all lobes fused at base; anterior lobes with one long row of slender spines along inner margin and one additional and parallel row of stronger spinules on anterior surface; posterior (central) lobe similar in shape and ornamentation to distal part of labrum but much smaller.

**Figure 4. F4:**
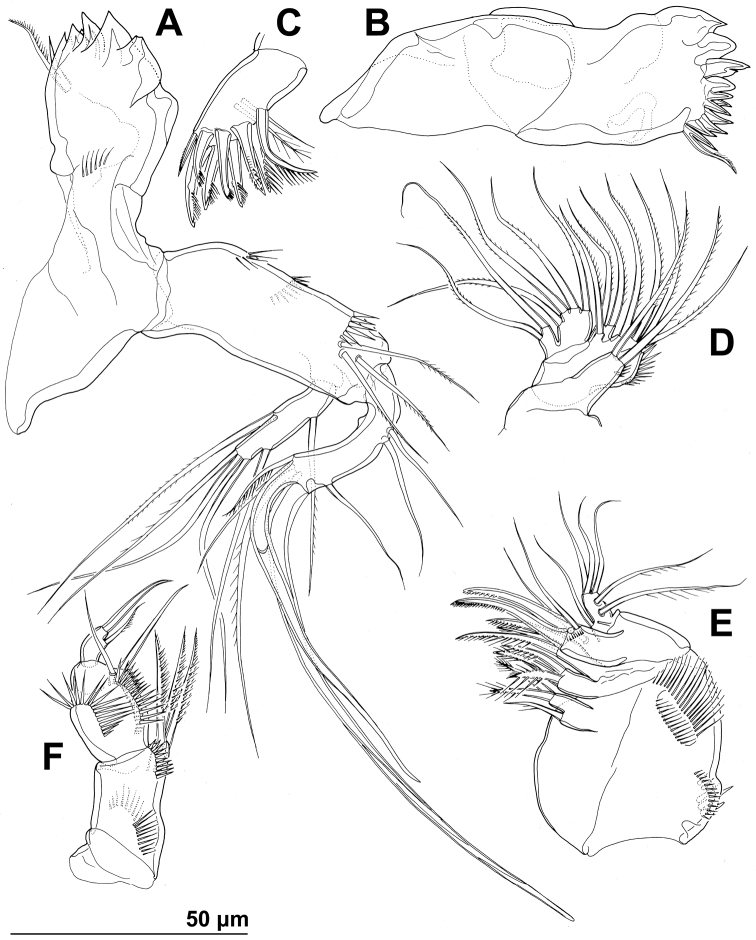
*Stenhelia pubescens* Chislenko, 1978, line drawings, female 3: **A** mandibula, posterior **B** mandibular coxa, anterior **C** maxillula, praecoxa arthrite, posterior **D** maxilular palp, posterior **E** maxilla, anterior **F** maxilliped, anterior.

Mandibula (Figs 2C, 4A) with wide cutting edge on relatively short coxa, with three strong bicuspidate teeth ventrally, eight smaller unicuspidate teeth dorsally, and single unipinnate dorsalmost seta; seta fused basally to neighbouring tooth and twice as long as it; only ornamentation on coxa short row of six slender posterior spinules. Palp biramous, comprising basis, one-segmented exopod, and one-segmented endopod. Basis with somewhat inflated central part, about 2.5 times as long as wide, with three slender but pinnate distal outer setae, and with three transverse rows of strong spinules, distalmost one with strongest spinules. Exopod 0.6 times as long as basis and less than half as wide, narrowest medially, curved back towards coxa and almost parallel with basis, with three lateral and five apical setae; all lateral and three apical setae slender, two apical setae strong and geniculate, longer one of them almost four times as long as exopod; two apical setae unipinnate, all other exopodal setae smooth. Endopod 0.8 times as long as exopod, 3.8 times as long as wide, with one inner, three apical, and two outer slender setae; inner seta bipinnate, proximal outer and inner apical setae unipinnate, others smooth.

Maxillula (Figs 2C, 4C, D) composed of praecoxa, coxa, basis, one-segmented endopod, and one-segmented exopod; endopod and exopod fused basally. Praecoxa large; arthrite rectangular, without spinules, with nine strong curved spines apically and subapically, all except ventralmost spine with dense tuft of distal spinules along convex margin; dorsalmost spine on praecoxal arthrite longest, ventralmost one shortest. Coxa with anterior arched row of short spinules, endite shorter than praecoxal arthrite, with three slender pinnate apical (on inner margin) setae. Basis wider and longer than coxa, with two endites, with dorsal row of strong spinules and three unipinnate setae on dorsal endite, and another three unipinnate setae on ventral endite. Endopod minute, rectangular, with four slender bipinnate apical setae. Exopod smaller than endopod, with two slender bipinnate apical setae.

Maxilla (Figs 2C, 4E) composed of large syncoxa, small basis and even smaller one-segmented endopod. Syncoxa with four rows of outer long spinules and with three endites; dorsal endite smallest, with one subapical and two apical strong pinnate setae; central and ventral endites slender, with three apical pinnate setae each, setae on ventral endite longest; two distal rows of spinules parallel on anterior surface, two proximal rows of spinules near outer margin, one on anterior, one on posterior surface, posterior distal surface smooth. Basis slightly larger than ventral endite of syncoxa, with anterior row of minute spinules, apically with two strong and geniculate, unipinnate spines, and two slender setae on ventral and posterior surfaces. Endopod much smaller than basis, twice as long as wide, with basal tubular pore, no spinules, with three lateral and three apical slender setae of similar length; two lateral setae unipinnate, others smooth.

Maxilliped (Figs 2C, 4F) prehensile, four-segmented, composed of coxa, basis, and two-segmented endopod. Coxa short, almost triangular, unarmed and unornamented. Basis largest and longest segment, about 1.8 times as long as wide and nearly five times as long as coxa, with one arched posterior row and two longitudinal anterior rows of slender spinules, with three strong unipinnate distomedial setae of about same length. First endopodal segment 0.8 times as long as basis but slightly wider, almost ovoid in shape, also with one posterior and two anterior rows of spinules but spinules much longer and stronger, with two smooth distomedial setae, one of them slightly longer and considerably stronger. Second endopodal segment minute, nearly rectangular, 1.6 times as long as wide, 0.4 times as long as first endopodal segment, unornamented, with apical strong prehensile smooth spine, and with subapical shorter and much more slender, unpinnate seta.

All swimming legs (Figs 1A, 2A) of similar size and long in comparison to body length, composed of small triangular and unarmed praecoxa, large rectangular and unarmed coxa, shorter and nearly pentagonal basis, slender three-segmented exopod, and slender three-segmented endopod; pair of legs joined by simple intercoxal sclerite.

**Figure 5. F5:**
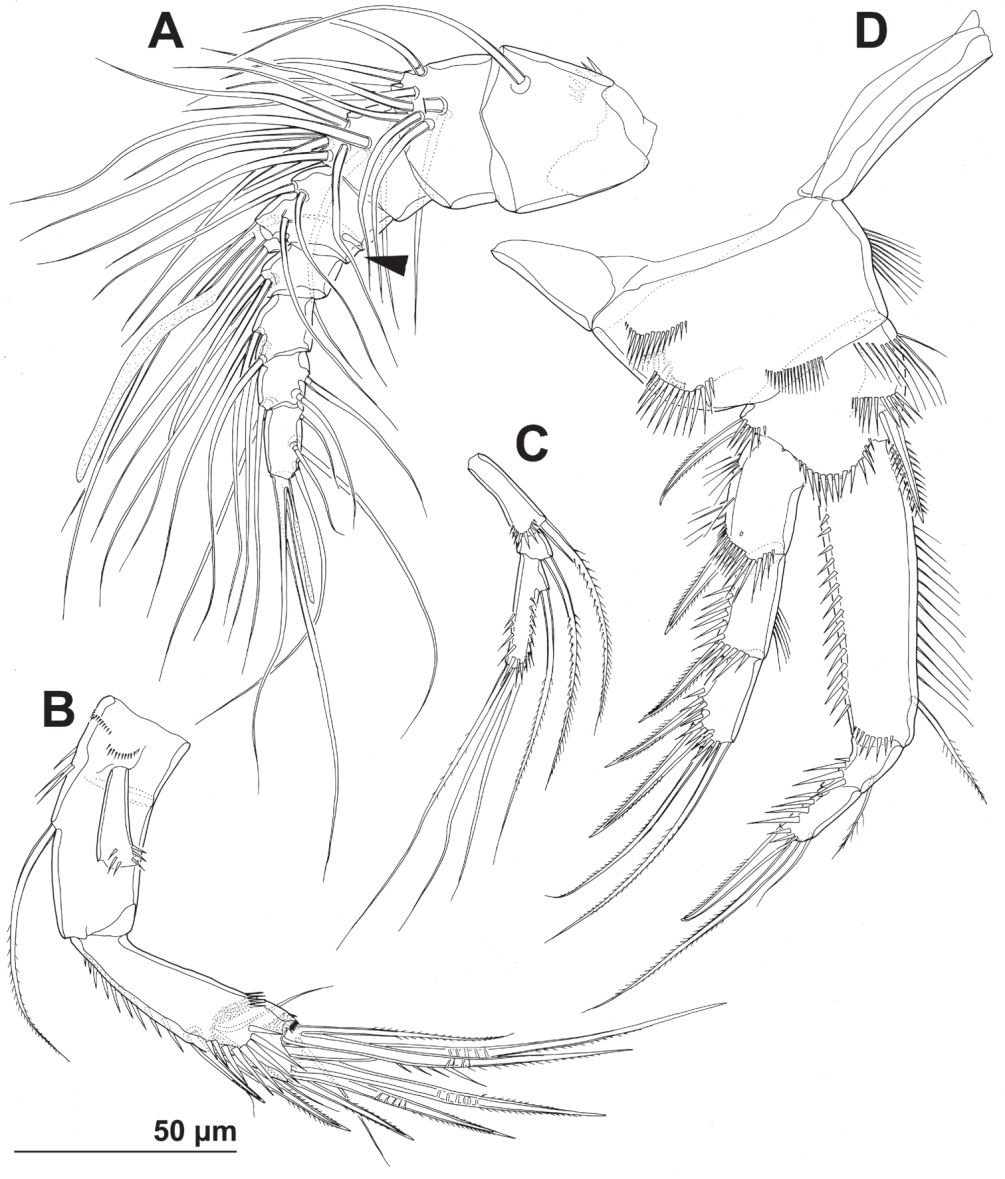
*Stenhelia pubescens* Chislenko, 1978, line drawings, female 3: **A** antennula, ventral **B** basis, endopod, and first exopodal segment of antenna, anterior **C** antennal exopod, anterior **D** first leg, anterior. Arrowhead indicates the presence of caudal suture on the fourth antennular segment.

First leg (Figs 1G, 2D, 5D) with smooth and short intercoxal sclerite, its distal margin nearly straight. Praecoxa longer than wide, longer than intercoxal sclerite but shorter than coxa, unornamented. Coxa 1.8 times as wide as long, with longitudinal row of long and slender inner spinules, three transverse rows of shorter but stronger anterior spinules, and two short rows of even smaller posterior spinules. Basis with one long strong and finely bipinnate outer spine, one shorter but stronger bipinnate inner spine, and four transverse rows of large anterior spinules (one at base of each spine, one at base of endopod, and one on proximal inner corner; latter with longest spinules). Exopod with all segments of similar length, each about twice as long as wide and with strong outer spinules and subdistally on anterior surface; first segment with anterior pore near distal outer corner; second segment with slender inner spinules; first two segments with single strong and finely bipinnate distolateral spine; third segment with two strong and finely bipinnate outer spines and two slender and finely bipinnate apical setae; apical setae not prehensile; length ratio of elements on third segment, starting from outer margin, 1 : 1.4 : 2 : 2.4. Endopod three-segmented, prehensile, about 1.4 times as long as exopod; first endopodal segment about as long as entire exopod and 3.3 times as long as wide, with slender and long inner spinules, shorter and stronger outer and anterodistal spinules, with single bipinnate inner seta, the latter slender and about 0.4 times as long as segment; second segment small, rhomboidal, slightly longer than wide and only one sixth of first segment’s length, with several strong anterodistal spinules, and single slender and bipinnate inner seta; latter about 1.6 times as long as segment; third segment about 2.5 times as long as wide and 1.4 times as long as second segment, with several strong inner spinules and three smaller antero distal spinules, with one slender inner seta, one strong and long apical seta, and another shorter and stronger outer apical spine; apical spine 1.7 times as long as third segment, half as long as apical seta, and 1.5 times as long as inner seta on third segment; longest seta on exopod and endopod of about same length.

**Figure 6. F6:**
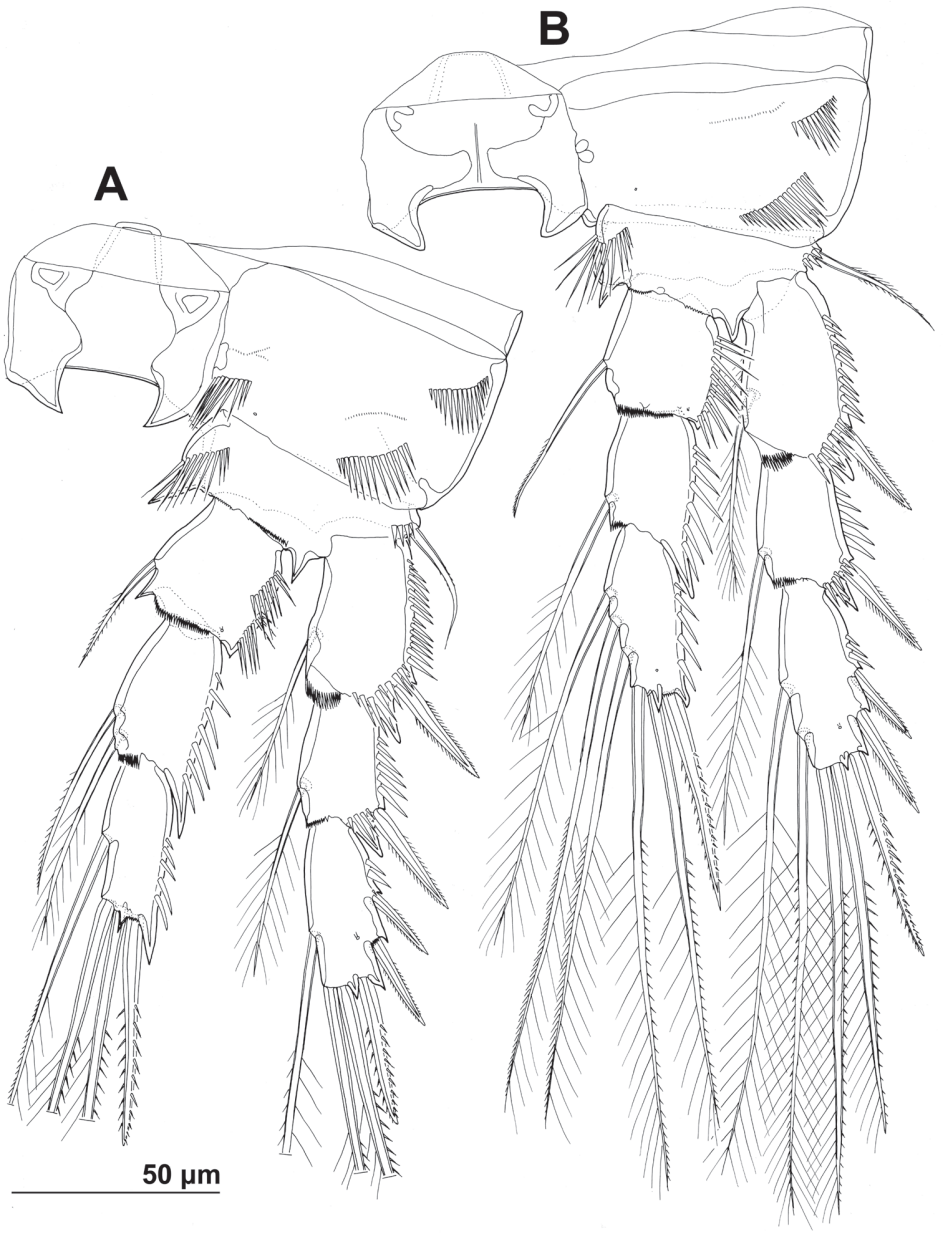
*Stenhelia pubescens* Chislenko, 1978, line drawings, female 3: **A** second leg, anterior **B** third leg, anterior.

Second leg (Figs 1G, 2E, 6A), intercoxal sclerite about as long as wide, unornamented, with two sharp and inwardly pointed distal processes. Praecoxa very short, unornamented. Coxa nearly 1.5 times as wide as long, with anterior pore near distomedial corner, three short rows of strong anterior spinules (one at distomedial corner, one near proximal outer corner, and one near distal outer corner), and two short rows of minute anterior spinules. Basis with nearly smooth (minute pinnules bearly visible), short and slender outer spine; inner distal corner produced into long and sharp process directed inwardly, another smaller distal process between exopod and endopod; with transverse row of long anterior spinules near inner margin, several smaller spinules ar base of outer spine, and discontinuous row of minute spinules at base of endopod. First exopodal segment widest, third segment slender and about 2.3 times as long as wide, 1.4 times as long as second segment, and about as long as first one; first and second segment with strong outer and anterodistal spinules and with distomedial frills, third segment with several outer strong spinules in proximal half and with anterior pore; first and second segments with single strong and finely bipinnate outer distal spine and slender bipinnate inner dista seta; third segment with three strong finely bipinnate outer spines, two apical strong bipinnate setae, and one slender bipinnate inner seta; inner apical seta on third segment longest, about 1.2 times as long as outer apical one, 2.4 times as long as third segment, and 2.7 times as long as outer distal spine; outer distal corner of first and second segment produced into small spiniform process. Endopod about as long as exopod; all segments of about same length, but progressively narrower from proximal to distal end, each with outer distal corner produced into strong spiniform process (first segment also with distomedial smaller process), and each with row of strong outer spinules, first two segments additionally with small distomedial frills, and first and third segments with anterior cuticular pore; armature consisting of single bipinnate inner seta on first segment, two pinnate slender inner setae on second segment, and one inner and three apical elements on third segment (probably outermost spine and two strong setae); seta on first segment about as long as segment, those on second segment about 1.4 times as long as segment, and those on third segment about twice as long as segment, except outer spine, which is about 1.4 times as long as segment. Two apical exopodal and endopodal setae each with shorter and stronger outer pinnules, inner setae on third exopodal and endopodal segments and proximal inner seta on second endopodal segment with shorter inner pinnules, all other bipinnate setae and spines with symmetrical pinnules.

Third leg (Figs 2E, 6B) similar to second leg, except for slightly less sharp processes on intercoxal sclerite, absence on distomedial row of strong spinules on coxa, smaller spiniform distomedial process on basis, two inner setae on third exopodal segment, one inner seta on second endopodal segment, and three inner seta on third endopodal segment; proximal inner seta on third endopodal and exopodal segment with long pinnules on both sides, distal inner seta on third exopodal segment with short pinnules on inner margin in addition to long ones, other setae and spines as in second leg.

**Figure 7.**
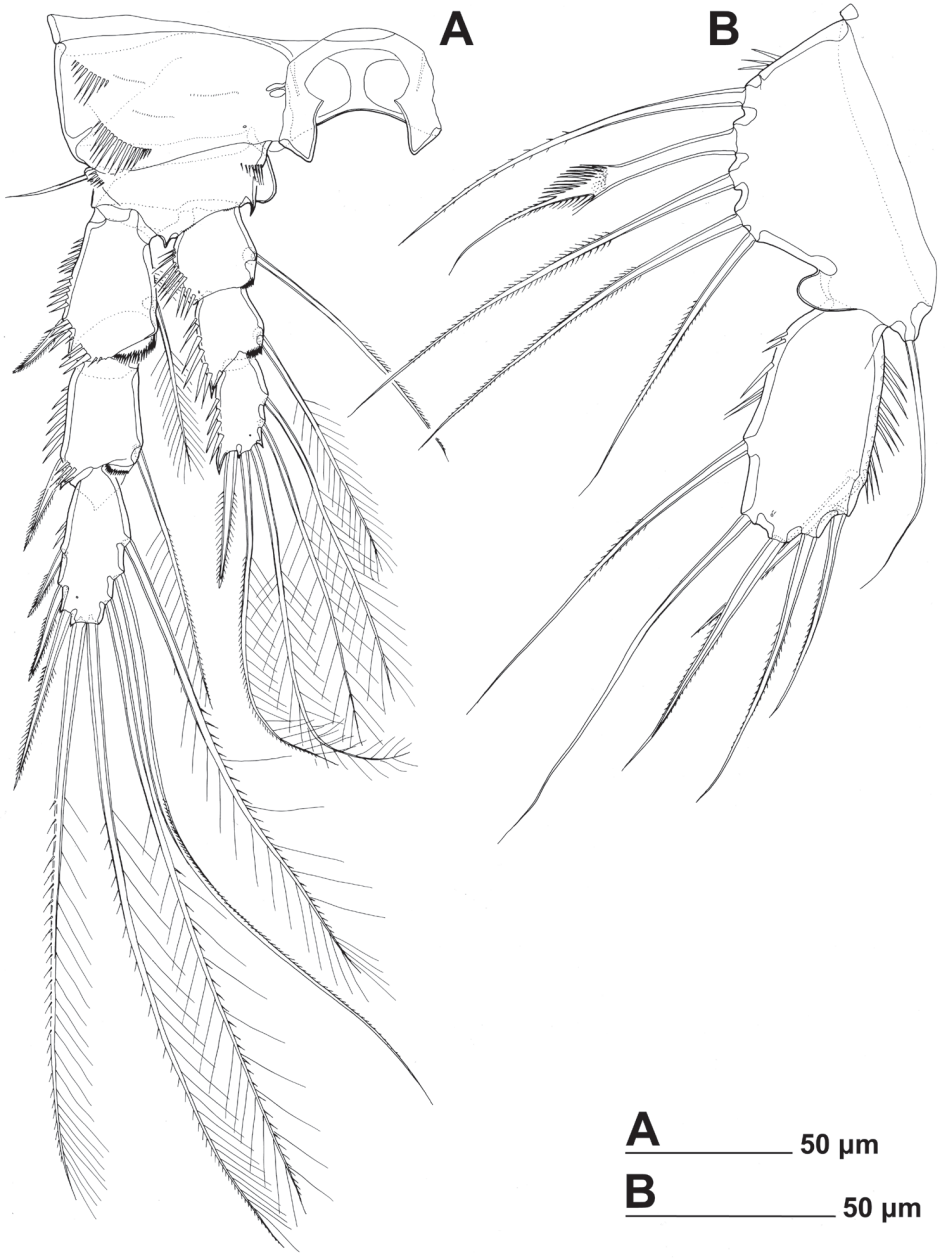
*Stenhelia pubescens* Chislenko, 1978, line drawings, female 3: **A** fourth leg, anterior **B** fifth leg, dissected and flattened, anterior.

Fourth leg (Figs 2E, 7A) relatively similar to third leg, but with endopod only about 0.6 times as long as exopod, with slightly shorter distomedial process on basis, much longer seta on first endopodal segment, only two inner setae on third endopodal segment, and three inner setae on third exopodal segment; central inner seta on third exopodal segment spiniform and characteristically curved inwards; all setae on third exopodal segment proportionately longer than in second or third leg.

Fifth leg (Figs 1D, 2F, 3B, F, 7B) composed of wide baseoendopod (fused basis and endopod) and much smaller and almost ovoid exopod, pair of legs joined by minute trapezoidal sclerite. Baseoendopod about 1.8 times as wide as long, more or less pentagonal, unornamented, with short and blunt process at base of exopod; outer basal seta slender and smooth, arising from short setophore, about 1.6 times as long as segment; endopodal lobe relatively narrow and short, more or less trapezoidal, not extending beyond proximal fifth of exopod, with five stout, bipinnate setae, their length ratio, starting from inner side, 1 : 0.8 : 1.2 : 1 : 0.8. Second endopodal seta from inner side with stout and smooth proximal half, characteristic transverse serrate comb near mid-length, and distal slender finely bipinnate whip; whip about as long as proximal part of seta. Exopod about 2.1 times as long as its maximum width, more or less ovoid, with narrower base than rest of it, with strong outer and inner spinules and single anterior pore close to distal margin, with six setae; innermost and second inner seta slender, others shorter and spiniform, second seta from inner side smooth, other setae bipinnate; length ratio of exopodal setae, starting from inner side, 1 : 1 : 1.4 : 1.4 : 0.6 : 0.6.

Sixth leg (Figs 2F, 3B, D, E) minute flap covering ventro-lateral genital aperture, mostly fused to somite, unornamented, with single short bipinnate seta near outer margin and one minute inner spine. Sixth legs seemingly joined on ventral side by fold-like suture which hides copulatory pores.

##### Variability.

Most morphological features in examined topotypes were conservative, including the sensilla and pores pattern on somites, and length ratio of different armature on appendages. The only significant form of morphological variability, except for the body length, was presence/absence of caudal suture on the fourth antennular segment (compare Figs 2B and 5A; arrowed in Fig. 5A) and the size of suture on the antennar allobasis indicating remnants of ancestral arthroidal membrane (Fig. 5B). We redescribe this species in order to show some previously unreported characters, so they can be compared with those of *Stenhelia taiae*. Differences from the original description of Chislenko (1978) are given in the Discussion section below.

#### Stenhelia taiae

Mu & Huys, 2002

http://species-id.net/wiki/Stenhelia_taiae

Figs 7 [Fig F8] [Fig F9] [Fig F10] [Fig F11] –12

##### Synonymy.

*Stenhelia taiae* sp. n. – Mu and Huys 2002, p. 187, Figs 10–13.

##### Type locality.

China, Bohai Sea, central region, sandy and muddy sediments at about 20 m depth, approximately 38.5°N, 120°E.

##### Specimens examined.

One female on one SEM stub (collection number NIBRIV0000232718), one female dissected on one slide (collection number NIBRIV0000232719), and two females destroyed for DNA sequences (GenBank accession nos. KF524885 & KF524884); all from South Korea, South Sea, Gwangyang Bay, sampling station 16, muddy sediments at about 10 m depth, 34.768889°N, 127.783806°E, 18 November 2012, leg. K. Kim.

##### Redescription of female.

**Figure 8. F8:**
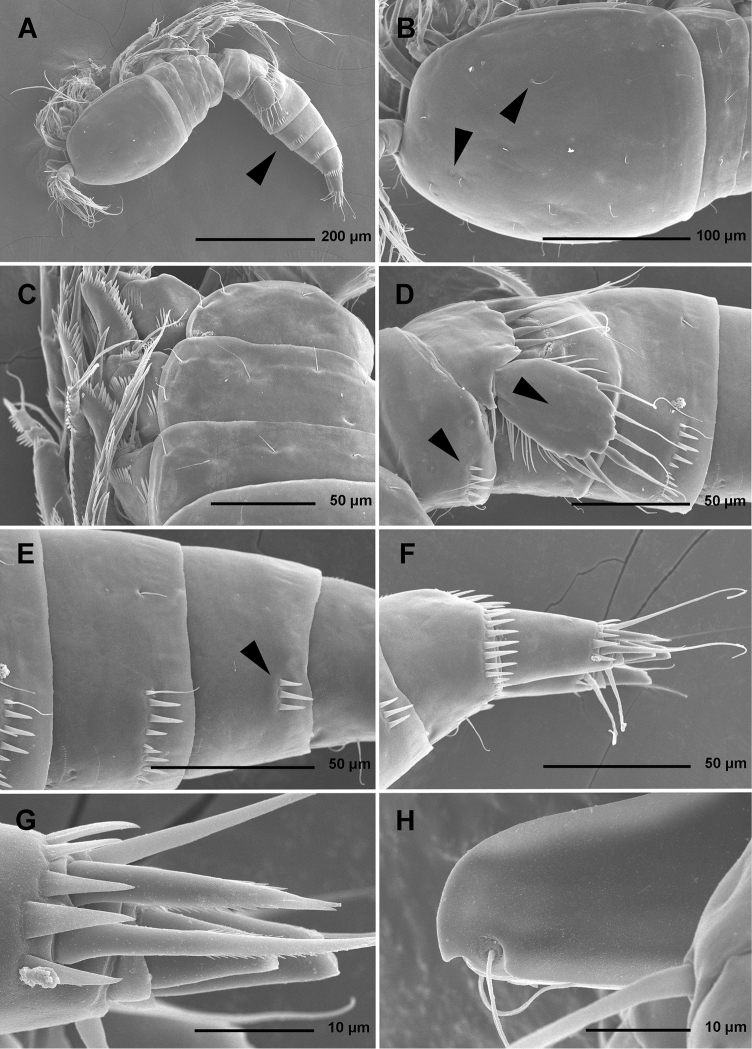
*Stenhelia taiae* Mu & Huys, 2002, scanning electron micrographs, female: **A** habitus, lateral **B** cephalothoracic shield, lateral **C** free thoracic somites, lateral **D** fifth pedigerous somite and genital double-somite, lateral **E** fourth and fifth urosomites, lateral **F** anal somite and caudal rami, lateral **G** posterior part of right caudal ramus, lateral **H** rostrum, lateral. Arrowheads indicate morphological characters different from those in *Stenhelia pubescens* Chislenko, 1978.

Body length from 565 to 578 μm (n = 4). Body segmentation, colour, nauplius eye, hyaline fringes, integument thickness and surface appearence as in *Stenhelia pubescens*, including very smooth integument on all somites and their posterior frills. Most somite ornamentation also similar to *Stenhelia pubescens*, and homologous pores and sensilla easy to establish. Habitus (Fig. 8A) slightly less robust, with proportionately longer urosome (arrowed in Fig. 8A), prosome/urosome length ratio less than 1.1, body length/width ratio about 3.1, cephalothorax 1.6 times as wide as genital double-somite.

Rostrum (Figs 8H, 10D) slightly longer and narrower in dorsal view than in *Stenhelia pubescens* (arrowed in Fig. 10D).

Cephalothorax (Fig. 8B) about 0.9 times as long as wide; comprising about 30% of total body length, with posterior lateral corner slightly more rounded than in *Stenhelia pubescens*. Surface of cephalothoracic shield ornamented as in *Stenhelia pubescens*, except one anterior pair of lateral sensilla absent (arrowed in Fig. 8B) and one additional pair of anterior pores present (also arrowed in Fig. 8B).

Pleurons of second to fourth pedigerous somites (Fig. 8C) without any difference in shape or ornamentation from those in *Stenhelia pubescens*.

First urosomite (Figs 8D, 10A, B) with three pairs of long sensilla, as in *Stenhelia pubescens*, but with one additional short row of strong lateral spinules (arrowed in Fig. 8D).

Genital double-somite (Figs 8D, 10A, B) shape and most ornamentation as in *Stenhelia pubescens*, except anterior dorsal pair of sensilla more widely spaced (arrowed in Fig. 10A), posterior ventral pair of sensilla closer to each other (arrowed in Fig. 10B), and no spinules in between posterior dorsal pair of sensilla.

Third urosomite (Figs 8E, 10A, B) as in *Stenhelia pubescens*, except no spinules in between posterior dorsal pair of sensilla.

Fourth urosomite (Figs 8E, 10A, B) as in *Stenhelia pubescens*, except with fewer lateral spinules (arrowed in Fig. 8E).

Anal somite (Figs 8F, 10A, B) similar to that in *Stenhelia pubescens*, but additional pair of dorsal pores present, posterior spinules smaller and less dense, and medial cleft slightly narrower.

Caudal rami (Figs 8F, G, 10A, C), much longer than in *Stenhelia pubescens* (arrowed in Fig. 10A), about 1.3 times as long as anal somite, cylindrical, 2.1 times as long as wide (ventral view), slightly divergent, and with space between them about one ramus width; ornamentation and armature as in *Stenhelia pubescens*, except inner apical seta much shorter and smooth (arrowed in Fig. 10C), and ventralmost lateral seta smooth and slender; posteroventral tubular pore also present, but ventral pore at base of lateral setae situated at two thirds of ramus length, not at midlength.

**Figure 9. F9:**
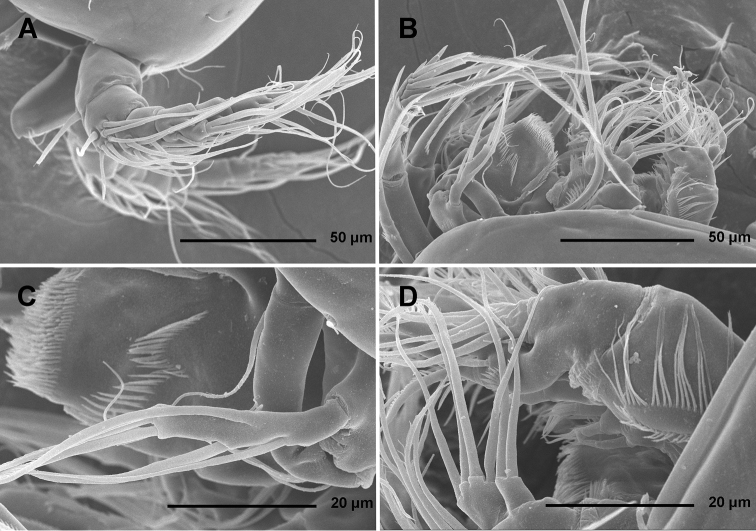
*Stenhelia taiae* Mu & Huys, 2002, scanning electron micrographs, female: **A** rostrum and antennulae, lateral **B** antenna and mouth appendages, lateral **C** mandibular palp and labrum, lateral **D** maxilla and part of maxillular palp, lateral.

Antennula (Fig. 9A), antenna (Fig. 9B), labrum (Figs 9C, 11A), paragnaths (Fig. 11B), mandibula (Fig. 9B, C), maxillula (Figs 9B, D, 11C), and maxilla (Figs 9D, 11D) as in *Stenhelia pubescens*.

**Figure 10. F10:**
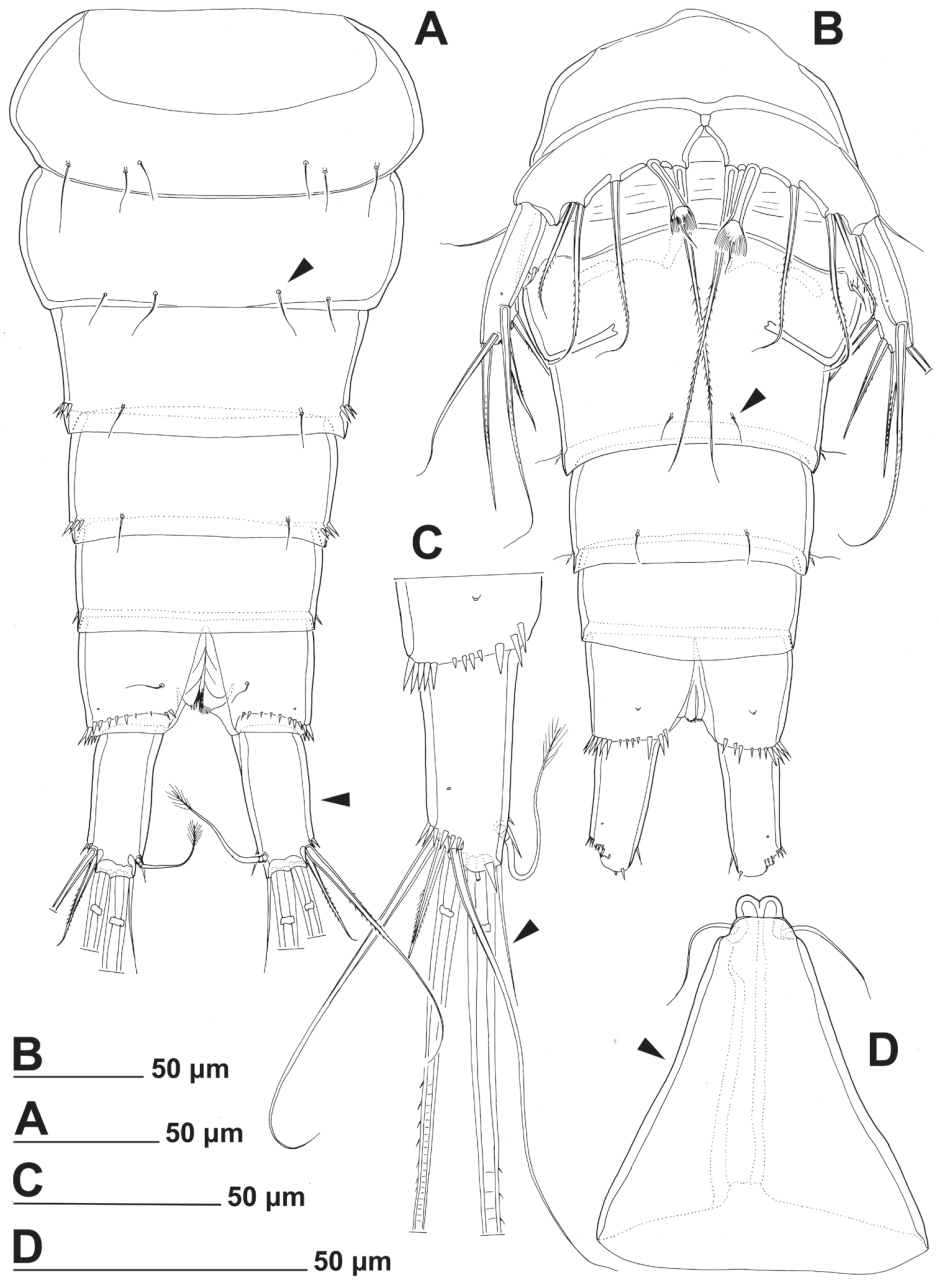
*Stenhelia taiae* Mu & Huys, 2002, line drawings, female: **A** urosome, dorsal **B** urosome, ventral (caudal rami armature omitted) **C** right caudal ramus, ventral **D** rostrum, dissected and compressed, dorsal. Arrowheads indicate morphological characters different from those in *Stenhelia pubescens* Chislenko, 1978.

**Figure 11. F11:**
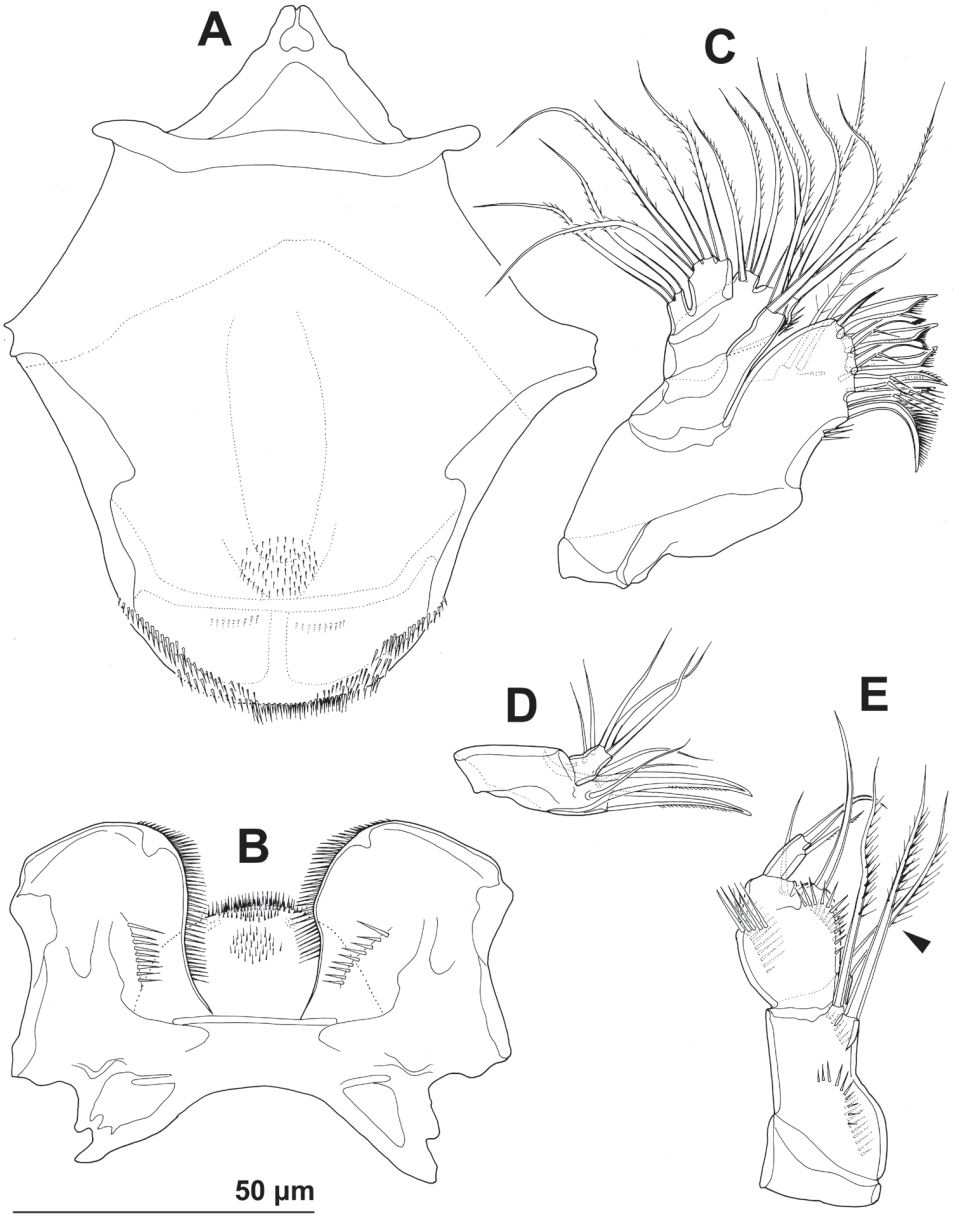
*Stenhelia taiae* Mu & Huys, 2002, line drawings, female: **A** labrum, posterior **B** paragnaths, anterior **C** maxillula, posterior **D** maxillar basis and endopod, posterior **E** maxilliped, posterior. Arrowhead indicates morphological character different from that in *Stenhelia pubescens* Chislenko, 1978.

Maxilliped (Fig. 11E) as in *Stenhelia pubescens*, except basal setae proportionately longer (arrowed in Fig. 11E) and apical endopodal spine proportionately shorter.

**Figure 12.**
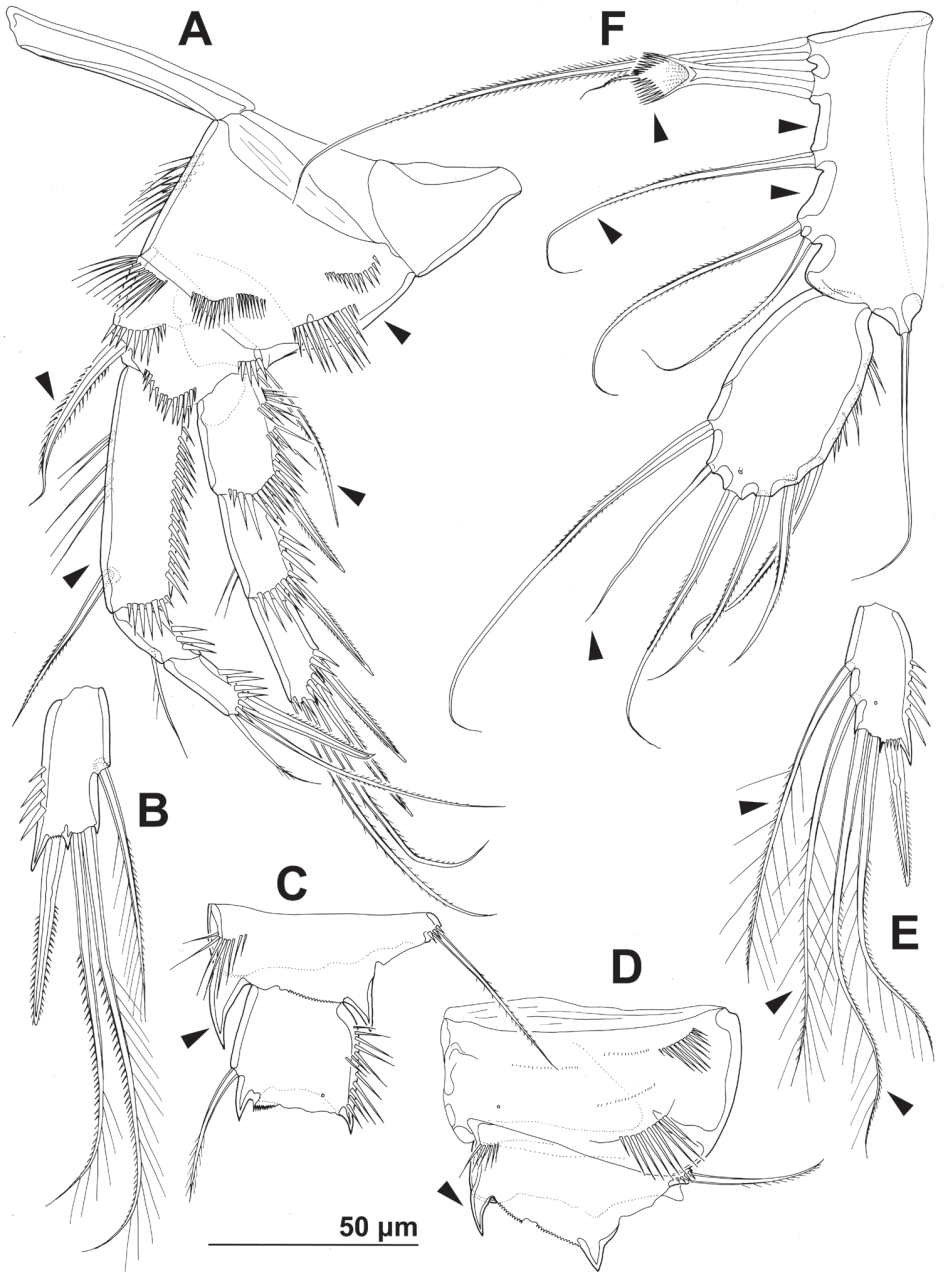
*Stenhelia taiae* Mu & Huys, 2002, line drawings, female: **A** first leg, anterior **B** third endopodal segment of second leg, anterior **C** basis and first endopodal segment of third leg, anterior **D** coxa and basis of fourth leg, anterior **E** third endopodal segment of fourth leg, anterior **F** fifth leg, dissected and flattened, anterior. Arrowheads indicate morphological characters different from those in *Stenhelia pubescens* Chislenko, 1978.

First leg (Figs 8A, C, 12A) as in *Stenhelia pubescens*, except first exopodal segment proportionately shorter, both basal spines proportionately longer, and coxa without posterior spinules (all four arrowed in Fig. 12A).

Second leg (Figs 8A, C, 12B) as in *Stenhelia pubescens*.

Third leg (Figs 8A, C, 12C) as in *Stenhelia pubescens*, except distomedial basal process slightly larger (arrowed in Fig. 12C).

Fourth leg (Figs 8A, 12D, E) as in *Stenhelia pubescens*, except distomedial basal process larger (arrowed in Fig. 12D), both inner setae on third endopodal segment with additional short pinnules (arrowed in Fig. 12E), and inner apical seta on third endopodal segment with short outer pinnules (arrowed in Fig. 12E).

Fifth leg (Figs 8D, 12F) segmentation, general shape, number of armature elements, and most ornamentation as in *Stenhelia pubescens*, except exopod proportionately shorter (arrowed in Fig. 8D), second endopodal seta from inner side shorter (arrowed in Fig. 12F), second and third endopodal seta from inner side shorter (both arrowed in Fig. 12F), and spaces between central endopodal seta and two neighbouring setae significantly wider (both arrowed in Fig. 12F). Distal whip on second endopodal seta much shorter than in *Stenhelia pubescens*, only about 0.35 times as long as proximal stout part of seta (including transverse serrate comb). Length ratio of endopodal setae, starting from inner side, 1 : 0.4 : 0.6 : 0.5 : 0.4. Length ratio of exopodal setae, starting from inner side, 1 : 0.5 : 0.7 : 0.5 : 0.5 : 0.6.

Sixth leg (Fig. 10B) as in *Stenhelia pubescens*.

##### Variability.

Most morphological features in the examined Korean specimens were extremely conservative, including the sensilla and pores pattern on somites, and length ratio of different armature on appendages. Except for the body length, the only other variable feature in the Korean population was the number of spinules on the inner margin of the fifth leg exopod (compare Figs 8D and 12F). We redescribe this species in order to show some previously unreported characters, so they can be compared with those of *Stenhelia pubescens*. Differences from the original description of Mu and Huys (2002) are given in the Discussion section below.

### Molecular results

DNA was extracted and the mtCOI fragment successfully PCR-amplified from 23 stenheliin copepod specimens (Table 2), belonging to eight different morpho-species. All the sequences were translated into protein using MEGA and were shown to have no evidence of stop codons, ambiguities or insertions–deletions indicative of non-functional copies of mtCOI. BLAST analyses of GenBank revealed that the obtained sequences are copepod in origin and not contaminants, and one of the GenBank COI sequences (JQ390578.1) from the species *Schizopera leptafurca* Karanovic & Cooper, 2012 was included in our phylogenetic analyses.

Average pairwise distances between morpho-species were found to be very high, with the lowest divergence (7.1%) between the Korean *Itostenhelia polyhymnia* Karanovic & Kim, 2014 and the Russian *Itostenhelia golikovi* (Chislenko, 1978) (Table 3). Second (10.1%) and third (16.9%) lowest divergences were found between *Stenhelia taiae* and *Stenhelia pubescens* and between *Stenhelia taiae* and *Willenstenhelia thalia* Karanovic & Kim, 2014, while those between all other taxa were in excess of 17%. These high divergence values are generally indicative of distinct species by comparison with other crustaceans (Lefébure et al. 2006) and other harpacticoid copepods (Karanovic and Cooper 2011a, 2012). Average pairwise distances among the four stenheliin genera were between 17% and 33.8%, indicating only a remote relationship, and are comparable to those among some well accepted canthocamptid and parastenocaridid genera (Karanovic and Cooper 2011a, b). They were certainly comparable to those between *Schizopera leptafurca* and the four stenheliid genera (from 19.9% to 37.6%), although the former belongs to a different subfamily of miraciid harpacticoids.

**Table 3.** Average pairwise maximum likelihood distances (TN model) among mtCOI sequences between each morpho-species (lower diagonal) and within morho-species (diagonal).

Species	1	2	3	4	5	6	7	8	9
1. *Wellstenhelia calliope*	-								
2. *Itostenhelia polyhymnia*	0.271	0.000							
3. *Wellstenhelia qingdaoensis*	0.267	0.228	-						
4. *Wellstenhelia clio*	0.202	0.328	0.245	-					
5. *Itostenhelia golikovi*	0.218	0.071	0.278	0.267	0.006				
6. *Willenstenhelia thalia*	0.285	0.201	0.291	0.338	0.181	0.008			
7. *Schizopera leptafurca*	0.302	0.241	0.376	0.344	0.270	0.199	-		
8. *Stenhelia taiae*	0.317	0.193	0.342	0.240	0.170	0.169	0.245	0.000	
9. *Stenhelia pubescens*	0.318	0.220	0.352	0.311	0.201	0.173	0.311	0.101	0.000

The highest divergences within morpho-taxa were those between eight specimens of *Willenstenhelia thalia* (0.8%), which all came from the same sampling station (St. 10), although collected on two separate occasions. Divergences between five specimens of *Itostenhelia golikovi* were about 0.6%. (Table 3). These are all indicative of intraspecific variability (Lefébure et al. 2006). Sequences of all other species where we had more than one specimen showed zero divergence, although being of different length (Table 2). The L-form of *Itostenhelia polyhymnia* shows no molecular divergence from the normal form of this species, despite their morphological difference in size and some cuticular ornamentation, although the amplified fragment was very short (Table 2).

**Figure 13.**
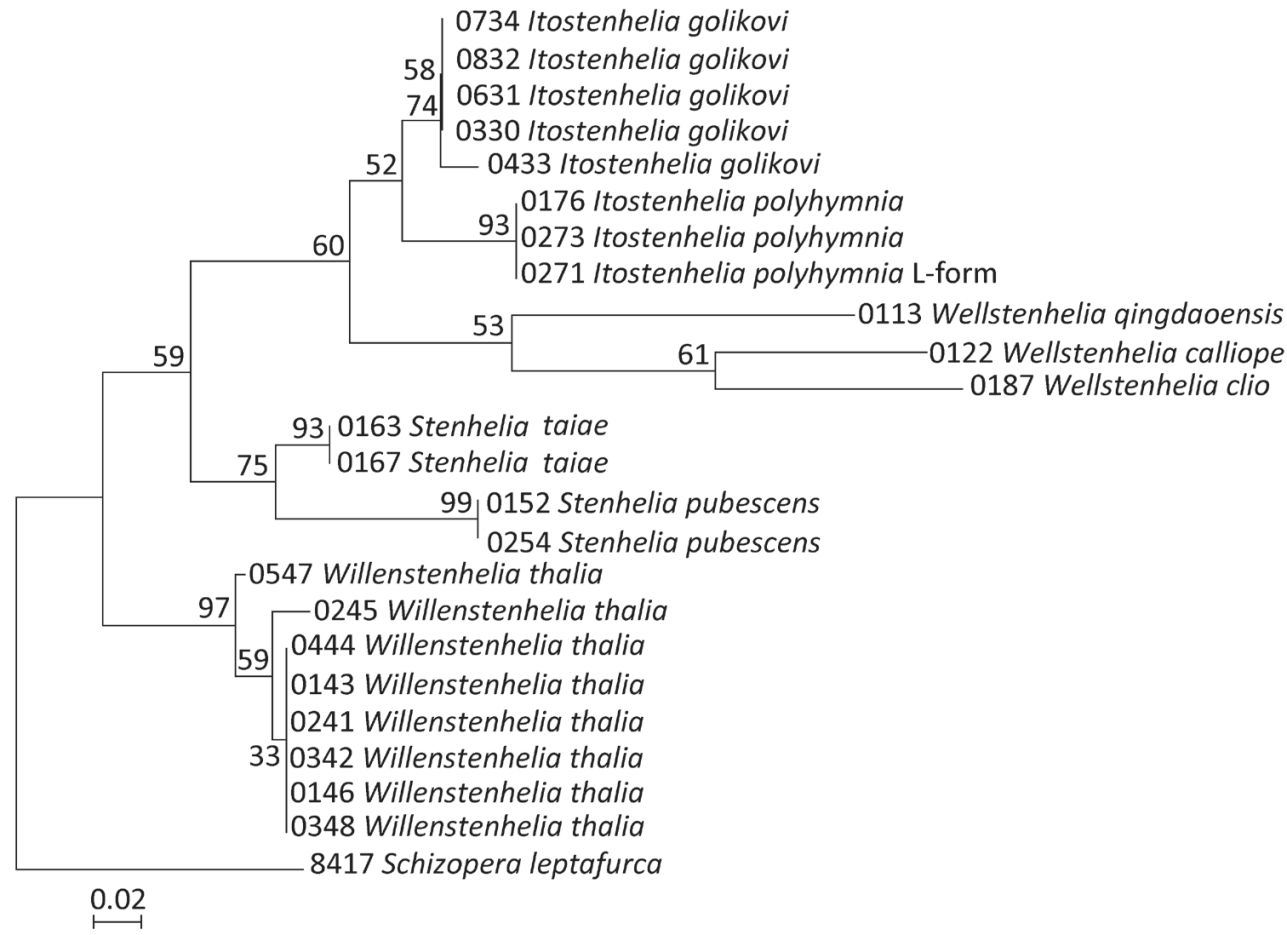
Maximum likelihood (ML) tree based on mtCOI sequence data of 23 stenheliin specimens from Gwangyang Bay (South Korea) and Posyet Bay (Russia), constructed using MEGA v 5.0.3 and an HKY+G model of evolution, with numbers on the branches representing bootstrap values from 500 pseudoreplicates. The tree is rooted with *Schizopera leptafurca* Karanovic & Cooper, 2012 from Western Australia. The cladogram is drawn to scale and the specimen codes correspond to those in Table 2.

All analyses (Fig. 13) supported the presence of at least nine highly divergent lineages and all five of the multisample lineages were supported with high bootstrap values (>74% for ML). The tree topology in our NJ analysis was the same as in the ML analysis (Fig. 13), except the bootstrap values were generally slightly higher. Our MP analysis resulted in two equally parsimonious trees, each 61 steps long, and their consensus also had a very similar topology to our ML tree, except that bootstrap values were generally slightly lower; also the terminal clade in *Willenstenhelia thalia* was not supported in our MP analysis, nor was the sister relationship between *Wellstenhelia calliope* Karanovic & Kim, 2014 and *Wellstenhelia clio* Karanovic & Kim, 2014 (instead a sister relationship was suggested between *Wellstenhelia qingdaoensis* (Ma & Li, 2011) and *Wellstenhelia clio*, but the bootstrap value for this clade was only 39%). Our previous morphological analyses (see Karanovic and Kim 2014) suggested that *Wellstenhelia clio* is more closely related to *Wellstenhelia calliope* than to *Wellstenhelia qingdaoensis* (see above), which is why we have more confidence in our ML analysis than in our MP analysis, and all further molecular results and subsequent discussion will refer to the former (Fig. 13).

All basal nodes are supported only by moderate bootstrap values (between 52% and 75%), which could be explained by the low phylogenetic resolution of the mtCOI gene in basal nodes of the trees, possibly due to saturation at third codon positions (Karanovic and Cooper 2012) and also by various lengths of the fragments amplified (see Table 2). Nevertheless, all four stenheliin genera were well defined. A sister group relationship of *Itostenhelia golikovi* and *Itostenhelia polyhymnia* has the lowest support (52%), yet these two morpho-species are only distinguishable by several settled morphological features, and so different from any other stenheliin analysed here that there is no doubt about their sister-species relationship (see Karanovic and Kim 2014). Another moderately supported lineage is that uniting the three *Wellstenhelia* species (53%), but it was recovered in all analyses despite each species being represented with a single sequence (Table 2); divergences between morpho-species are much higher than in the genus *Itostenhelia*, which is in complete accordance with previously observed morphological evidence. There is also a strongly supported sister group relationship of *Stenhelia pubescens* and *Stenhelia taiae* (bootstrap support 75%). Genera *Itostenhelia* and *Wellstenhelia* form a moderately supported clade (60%), with a similar level of support suggested for the lineage formed by these two genera and the genus *Stenhelia*. All our analyses showed *Willenstenhelia* as a sister group to all other stenheliins, suggesting only a remote relationship; although this was not apparent from the divergence values (Table 3), it is strongly supported by the previously studied morphological data (see Karanovic and Kim 2014).

## Discussion

**Phylogenetic implications.** Our phylogenetic analysis (Fig. 13) resulted in demonstrating a polyphyly of the genus *Delavalia* Brady, 1869, as postulated by Mu and Huys (2002), because all species described or redescribed in this paper would traditionally be (and *Wellstenhelia qingdaoensis* and *Itostenhelia golikovi* indeed used to be) classified as belonging to this genus. However, the position of the genus *Stenhelia* deep inside this stenheliin group suggests that the two-segmented endopod of the first leg must have originated independently at least in *Willenstenhelia* and *Itostenhelia*/*Wellstenhelia*. The simplicity of the genus-group division based on this morphological character alone was recently demonstrated in the closely related subfamily Diosaccinae Sars, 1906 by Karanovic and Cooper (2012), also based on the combined molecular and morphological approach. A more robust phylogeny of miraciids in general and stenheliins in particular would have to be based on a wider taxon sampling and more genes (including some slower evolving nuclear ones, such as 18S; see Karanovic and Krajicek 2012a), but the initial congruent data between morphology and genes (Karanovic and Kim 2014) are encouraging for this group of harpacticoid copepods with very few species being resampled after their initial description and many with even their types lost. Also encouraging was the fact that the topology of our trees changed very little depending on the method used (with essentially no difference between NJ and ML analyses), which may suggest that our data are robust (i.e. phylogenetically informative), despite a relatively short segment of the mtCOI gene being amplified (especially in some specimens; see Table 2).

The smallest average divergence values in mtCOI gene (Table 3) were observed between two allopatric (Korea/Russia) species pairs: *Itostenhelia polyhymnia*/*Itostenhelia golikovi* and *Stenhelia taiae*/*Stenhelia pubescens* (7.1% and 10.1% respectively). Average divergence values between all sympatric Korean stenheliins were very high (all in excess of 16.9%), which suggests a long independent evolutionary history. This is also reflected in their numerous morphological differences (Karanovic and Kim 2014). To us this suggests a potential for niche partitioning with minimal competition for resources, and is very similar to some recently observed examples of sympatric Australian diosaccins (Karanovic and Cooper 2012). It means that multiple colonisations are a better model for explaining this unprecedented diversity in a small Korean bay than is an explosive radiation, despite the fact that surrounding areas do not hold a high diversity currently. However, without any fossil record we can only guess what the diversity of this group in East Asia was historically. Anthropogenic translocation may also be a contributing factor, as for some other copepod groups (see Karanovic and Krajicek 2012a), and especially through ships’ ballast water discharge (Reid and Pinto-Coelho 1994; Lee 1999) or ship’s hull biofouling. However, this is just a speculation at this stage, but the presence of *Willenstenhelia minuta* (A. Scott, 1902) in the Suez Canal in Egypt (Gurney 1927) is a sign that these animals are easily dispersed even in artificial habitats.

**Micro-characters in harpacticoid taxonomy.** Lang (1965) was the first to start paying special attention to somite ornamentation in harpacticoids, and to use it as a diagnostic character in species descriptions and delineations, especially in regard to the spinules pattern on urosomites. Pores and sensilla pattern have not been used in harpacticoid taxonomy until recently, despite their usefulness being demonstrated in distinguishing closely related species of both calanoid (Fleminger 1973; Mauchline 1977; Malt 1983; Koomen 1992) and cyclopoid copepods (Strickler 1975; Baribwegure and Dumont 1999; Baribwegure et al. 2001; Baribwegure and Mirabdullayev 2003; Alekseev et al. 2006; Karanovic and Krajicek 2012a; Karanovic et al. 2012). Initial studies in harpacticoids showed different results in different groups. In the freshwater family Parastenocarididae Chappuis, 1940 a combined morphological and molecular approach showed that spinules ornamentation on urosomites can be used to distinguish between closely related sister species (Karanovic and Cooper 2011a); however, sensilla pattern seems to be extremely conservative within certain lineages (Karanovic and Cooper 2011a; Karanovic et al. 2012; Karanovic and Lee 2012), thus being potentially useful in reconstructing their phylogenetic relationships. Several examined species of the parastenocaridid genus *Proserpinicaris* Jakobi, 1972 all have 45 pairs of sensilla on their body (Karanovic et al. 2012), while those of the genus *Parastenocaris* Kessler, 1913 have only 40 pairs of sensilla (Karanovic and Lee 2012). Their homologisation seems to be relatively uncomplicated, and may prove useful in future revisions of this problematic family. In the family Ameiridae Monard, 1927, a study of several marine species showed a greater diversity in the sensilla and pores pattern even between closely related species (Karanovic and Lee 2012), suggesting them as very useful characters for species delineation. Predictably, their homologisation proved to be much more difficult. Large differences in the sensilla and pores pattern were observed between the stenheliin genera *Itostenhelia*, *Wellstenhelia* and *Willenstenhelia*, but very few between closely related species and with almost non-existant intraspecific variability (Karanovic and Kim 2014).

In this study, one of our aims was to examine pores and sensilla pattern of the two closely related *Stenhelia* congeners. Differences involved not just relative positions of some pores and sensilla, but also a complete absence of some. Cephalothoracic shield has one sensilla pair less and one pore pair more in *Stenhelia taiae* than in *Stenhelia pubescens* (compare Figs 1B and 8B). Genital double-somite in *Stenhelia taiae* has the ventral posterior pair of sensilla less widely spaced and the dorsalmost anterior pair of sensilla more widely spaced than in *Stenhelia pubescens* (compare Figs 3A, B and 10A, B). Finally, the anal somite in *Stenhelia pubescens* lacks the dorsal pair of pores (compare Figs 3A and 10A). Differences between these two species in the cuticular pores and sensilla pattern are no fewer than differences in the more tradidionally used macro-morphological characters, such as the length of caudal rami (compare Figs 3A and 10A), shape and armature proportions of the fifth leg (Figs 7B and 12F), several differences in shape and ornamentation of the swimming legs (Figs 5D, 6B, 7A and 12A, C, D, E), and spinular ornamentation of the urosomites (Figs 1D, E and 8D, E). This is all very surprising given their relatively low divergence values in the mtCOI gene of only 10.1% (see Table 3).

Almost all pores and sensilla can be homologised in these two species without many problems, suggesting a potential use of these structures in future phylogenetic reconstructions of harpacticoid copepods. However, many more families would have to be studied before this could happen. Even so, these preliminary studies in three of the four largest harpacticoid families (Boxshall and Halsey 2004) suggest that these characters hold a huge potential for phylogenetic studies, especially where traditional macro-morphological characters are extremely conservative (family Parastenocarididae, for example) or where they show a great number of homoplastic changes (in most subterranean taxa; see Karanovic and Hancock 2009; Karanovic 2010).

**Discrepancies between original descriptions and redescriptions.** Careful examination of our topotypes of *Stenhelia pubescens* revealed a number of morphological differences from the original description by Chislenko (1978). We did not examine the types deposited at the Zoological Museum in St. Petersburg, because they are in bad condition, as are most specimens deposited there by Chislenko (pers. comm. Dr Elena Chertoprud, Moscow State University). We were able to check this for the holotype of *Enhydrosoma intermedia* Chislenko, 1978 for example (see Kim et al. in press). Most importantly, we confirm that the second innermost seta on the fifth leg endopod is transformed (see Fig. 3F), with a characteristic transverse posterior serrate comb near mid-length. This was inconclusive in the original description, and it is one of the major synapomorphies of the genus *Stenhelia* as redefiend by Mu and Huys (2002). Other major differences between the original description and our redescription include the number of setae on the antennula, antenna, and maxilliped, and it is more probable that they are observational errors on Chislenko’s part than intraspecific variability. For example, his drawings show only 6.8.2.3.3 setae on the second to sixth antennular segments, while in reality that formula is 11.9.6.3.4 (see Figs 2B, 5A). Similarly, he probably overlooked two very slender setae on the ultimate endopodal segment of antenna (Fig. 5B) and one on the second endopodal segment of maxilliped (Fig. 4F). The latter is present in most stenheliins that have been studied in detail. Expectedly, there are numerous other smaller differences in the ornamentation of somites and appendages, just because they were not studied in detail or not studied at all by Chislenko (for example, intercoxal sclerites of the swimming legs, sensilla and pore pattern of most somites, etc.). Two other smaller differences are worth mentioning: long setules on the caudal rami armature and a curved seta on the third exopodal segment of the fourth leg. The former are limited to distal tips of the innermost apical and longest lateral caudal setae (Figs 2G, 3A, B), and are not present along the entire length of the armature elements as illustrated by Chislenko (1978, p. 194, fig. 9.2). It is possible that he interpreted some filamentous bacterial colonies as long setules, as these can be seen in several places on our specimens (see Fig. 1C). He has similarly mistaken a bacterial filamentous growth for a slender seta on the maxilliped in his description on *Enhydrosoma intermedia* (see Kim et al. in press). The curved seta on the fourth leg exopod (Fig. 7A) could have been interpreted as a mounting artefact by Chislenko (1978), who drew this element as all other exopodal setae. Differences between specimens that we examined and those examined by Chislenko in the shape of urosome and proportions of the genital double-somite are clearly a consequence of different compression during mounting. We do not think any of the above mentioned differences could be attributed to intraspecific variability, as we examined topotypes and found very little variability among our specimens. In all our samples from the Russian Far East *Stenhelia pubescens* and *Itostenhelia golikovi* were the only two stenheliins, so there is no possibility that the topotypes we redescribed here belong to a different species than specimens described by Chislenko (1978).

As for the differences between Korean and Chinese populations of *Stenhelia taiae*, they are all minor and some could possibly be contributed to geographic intraspecific variability. We did not examine the types of this species either, but the original drawings of Mu and Huys (2002) are recent, very skilful and detailed, and most differences involve minute details of ornamentation of somites. For example, we could not verify the presence of ventrolateral pores on the genital double-somite and on the third urosomite, despite making high resolution SEM photographs of this area (see Fig. 8D, E). Similarly, Mu and Huys (2002) reported two anterior lateral sensilla on the cephalothoracic shield, as in *Stenhelia pubescens* (see Fig. 1B), but we could only observe one sensilla in that spot (arrowed in Fig. 8B). It is more plausible that these difference are a result of intraspecific variability than of observational errors, as most other sensilla and pores are in exactly the same spot, inlcluding closely spaced ventral posterior sensilla on the genital double-somite, ventral pores on the anal somite, and tubular pore on the posterior ventral margin of the caudal ramus (Fig. 10B, C). It is, however, quite certain that Mu and Huys (2002) overlooked several lateral sensilla and pores on the cephalothoracic shield, especially along the ventral margin, as these are present in all harpacticoids examined in detail so far, and in all stenheliins examined here and elsewhere (see Karanovic and Kim 2014). Unfortuantely, some of them are actually only visible from ventral side in some taxa (see Fig. 2), and sometimes filametous bacteria and other epiphytes can be mistaken for sensilla (compare, for example, our Figs 1C and 8C).

**Morphology and phylogeny of*Stenhelia*.** Major synapomorphy of the eigth species currently recognised as members of this genus, as redefiend by Mu and Huys (2002), is the transformed second innermost seta on the female fifth leg endopod. The condition of this character was unknown in *Stenhelia pubescens* before our redescription, but we confirm its presence above (see Figs 3F, 7B). Monophyly of this genus has also been supported in our molecular analyses (Fig. 13). Mu and Huys (2002) recognised two major groups of species in the genus based on the number of setae on the third endopodal segment of the third leg. The first group includes the type species *Stenhelia gibba* and two other congeners: *Stenhelia curviseta* and *Stenhelia proxima*. They all have two inner setae on that segment, and are distributed in the Northern Atlantic and the Mediterranean Sea (Lang 1948; Apostolov and Marinov 1988), but differ markedly in the length ratio of armature elements on the fifth leg, as well as in the relative length of the first endopodal segment of the first leg. The other group has three inner setae on the third endopodal segment of the third leg and contains one species from the Atlantic Ocean (*Stenhelia divergens*) and four from the Northern Pacific (*Stenhelia peniculata*, *Stenhelia pubescens*, *Stenhelia sheni*, and *Stenhelia taiae*). Mu and Huys (2002) noticed that the only Atlantic species in the second group can be distinguished from its Pacific congeners by the shape of the first leg endopod, and they also provided a useful key to species. It should be noted that the second group of species is based on a plesiomorphic character state and that the condition of this character in the first group could be homoplastic. It is quite possible that one of three inner setae can be reduced convergently, and it does not even have to be the same seta to produce the apparent two-inner-setae condition (for some examples of this see Karanovic and Hancock 2009; Karanovic et al. 2013). Morphology, however, did suggested that the three East Asian species are quite similar in comparison to other congeners (Mu and Huys 2002), and testing that hypothesis was one of major aims of our study. Our reconstructed molecular phylogeny (Fig. 13) confirmed this hypothesis at least for two East Asian species, with remarkably low divergence values (Table 3) between *Stenhelia pubescens* and *Stenhelia taiae* specimens. The divergence value of 10.1% in the mtCOI gene is low not only in comparison with other crustaceans (see Lefébure et al. 2006) but also in comparison with sister-species with parapatric distribution and niche partitioning from the closely related subfamily Diosaccinae (see Karanovic and Cooper 2012), where these values were in excess of 15%. Even lower divergence rates were observed between two sister species of the genus *Itostenhelia* (see Karanovic and Kim 2014), which may imply that either this gene evolves more slovely in stenheliins or that the rate of speciation is higher. Similarly low divergence rates were found recently between several Western Australian species of the parastenocaridid genus *Kinnecaris* Jakobi, 1972 (see Karanovic and Cooper 2011a), which are all short range endemics and allopatric in distribution, with only minute morphological differences, and thus probably a product of a relatively recent speciation. *Stenhelia pubescens* and *Stenhelia taiae* are also allopatric species, of course, but their numerous morphological differences stand in stark contrast to their low divergence rates in the mtCOI gene.

## Supplementary Material

XML Treatment for Stenhelia pubescens

XML Treatment for Stenhelia taiae
